# Characterizing neuroinvasion and neuropathology of SARS-CoV-2 by using AC70 human ACE2 transgenic mice

**DOI:** 10.3389/fmicb.2024.1455462

**Published:** 2024-09-24

**Authors:** Jason C. Hsu, Panatda Saenkham-Huntsinger, Pinghan Huang, Cassio Pontes Octaviani, Aleksandra K. Drelich, Bi-Hung Peng, Chien-Te K. Tseng

**Affiliations:** ^1^Department of Biochemistry, Cell & Molecular Biology, The University of Texas Medical Branch, Galveston, TX, United States; ^2^Department of Microbiology & Immunology, The University of Texas Medical Branch, Galveston, TX, United States; ^3^Department of Neuroscience, Cell Biology, & Anatomy, The University of Texas Medical Branch, Galveston, TX, United States; ^4^Department of Pathology, The University of Texas Medical Branch, Galveston, TX, United States

**Keywords:** SARS-CoV-2, neuropathology, trigeminal, neuroinvasion, COVID-19

## Abstract

COVID-19 presents with a plethora of neurological signs and symptoms despite being characterized as a respiratory disease, including seizures, anxiety, depression, amnesia, attention deficits, and alterations in consciousness. The olfactory nerve is widely accepted as the neuroinvasive route by which the etiological agent SARS-CoV-2 enters the brain, but the trigeminal nerve is an often-overlooked additional route. Based on this consensus, we initially conducted a pilot experiment investigating the olfactory nerve route of SARS-CoV-2 neuroinvasion via intranasal inoculation in AC70 human ACE2 transgenic mice. Notably, we found that the trigeminal ganglion is an early and highly efficient site of viral replication, which then rapidly spread widely throughout the brain where neurons were primarily targeted. Despite the extensive viral infection across the brain, obvious evidence of tissue pathology including inflammatory infiltration, glial activation, and apoptotic cell deaths were not consistently observed, albeit inflammatory cytokines were significantly induced. However, the expression levels of different genes related to neuronal function, including the neurotransmitter dopamine pathway as well as synaptic function, and markers of neuronal damage were altered as compared to mock-infected mice. Our findings suggest that the trigeminal nerve may serve as a neuroinvasive route complementary to the olfactory nerve and that the ensuing neuroinvasion presented a unique neuropathological profile. This study provides insights into potential neuropathogenic mechanisms utilized by coronaviruses.

## Introduction

Over four years now since its emergence in Wuhan, China (World Health Organization, 2020). Coronavirus Infectious Disease-2019 (COVID-19) has infected over 700 million people in total across the globe, and killed over 6.9 million of them (Dong et al., 2020). The etiological agent of COVID-19 is a member of the family *Coronaviridae* in the *Betacoronavirus* genus designated “Severe Acute Respiratory Syndrome-Coronavirus-2” or “SARS-CoV-2” (Coronaviridae Study Group of the International Committee on Taxonomy of Viruses, 2020). Despite being characterized as a primarily respiratory disease, COVID-19 importantly also exhibits neurological symptoms that range from the mild, such as loss of sense of smell (anosmia) and taste (ageusia) as well as headache and fatigue, to the severe, such as strokes and seizures, as well as neuropsychiatric disorders including delirium, anxiety, depression, psychosis, memory loss (amnesia), and attention deficits (Baig and Sanders, 2020; Bauer et al., 2022; DosSantos et al., 2020; Huang et al., 2020; Solomon et al., 2020; Solomon, 2021; Song et al., 2021; Arbour et al., 2000; Desforges et al., 2014); with the plethora of neurological symptoms, it is undeniable that there is neurological system involvement in the pathogenesis of COVID-19. After having caused much death and illness globally, COVID-19 continues to be a public health issue in the form of lingering neurological symptoms and post-acute sequelae (Al-Aly et al., 2021; Proal and VanElzakker, 2021). Yet, despite great strides in the research effort in recent years, many of the neuropathogenic mechanisms of COVID-19 continue to elude elucidation, which would help in informing treatment of the neurological disorders that arise after disease onset.

One such neuropathogenic mechanism that still merits investigation is the routes of neuroinvasion taken by SARS-CoV-2. There is plentiful evidence to suggest SARS-CoV-2 has significant neuroinvasive potential (Baig and Sanders, 2020; Bauer et al., 2022; DosSantos et al., 2020; Song et al., 2021; Arbour et al., 2000; Desforges et al., 2014; Jeong et al., 2022; Morris and Zohrabian, 2020; Natoli et al., 2020; Paniz-Mondolfi et al., 2020). SARS-CoV-2 viral proteins and RNA have been detected within the brains of both COVID-19 patients and SARS-CoV-2-infected mice, leading to associated tissue pathology, such as microglial activation and immune infiltrates (Song et al., 2021; Jeong et al., 2022; Meinhardt et al., 2021; Jeong et al., 2022). Furthermore, neural tissue cells, including neurons and glia, do express, albeit at low levels, angiotensin-II-converting enzyme (ACE2) as well as transmembrane serine protease 2 (TMPRSS2), which have been identified as the main host enzymatic cofactors determining viral entry into permissive host cells (Hoffmann et al., 2020; Hoffmann et al., 2020; Chen et al., 2021). Although at least eight routes of neuroinvasion have been hypothesized to be utilized by SARS-CoV-2 (Baig and Sanders, 2020; Bauer et al., 2022; DosSantos et al., 2020; Song et al., 2021; Arbour et al., 2000; Desforges et al., 2014; Jeong et al., 2022; Morris and Zohrabian, 2020; Natoli et al., 2020; Paniz-Mondolfi et al., 2020), the current scientific consensus is the direct olfactory nerve is the main route of SARS-CoV-2 neuroinvasion, owing to the immediate exposure of the nerve endings to the outside atmosphere and short lengths of the nerves leading to the close proximity of the olfactory bulb (OB) to the external environment (Meinhardt et al., 2021). Nevertheless, an oft-understudied route of neuroinvasion that should be considered here in the context of SARS-CoV-2 infection is the trigeminal nerve. Most well-characterized in studies involving human herpesvirus infections, particularly serotypes 1/2/3/6 (HHV-1/2/3/6) (Theil et al., 2003; Beier, 2021; Shimeld et al., 2001; Ptaszyńska-Sarosiek et al., 2019), the trigeminal nerve route of neuroinvasion is a particularly attractive route of neuroinvasion because the trigeminal neurons are directly connected to the brainstem at the pons while the nerve terminals still end in very close proximity to the external environment, only being separated by nasal epithelial cells, which have been reported to be susceptible to infection by SARS-CoV-2, thereby bypassing the blood–brain barrier (BBB) (Meinhardt et al., 2021; Kamel and Toland, 2001; Schaefer et al., 2002; Tremblay and Frasnelli, 2018; Romano et al., 2019).

There have been a few studies that seemingly confirm the trigeminal nerve route of neuroinvasion by SARS-CoV-2. Early in the pandemic, a study reported the detection of SARS-CoV-2 viral genome copies within the human trigeminal ganglion (TG) while also reporting the detection of viral proteins in the human olfactory epithelium (OE) and OB (Meinhardt et al., 2021). Similarly, a different study in K18 human ACE2 (hACE2) transgenic mice also reported the detection of SARS-CoV-2 viral genomes and infectious virions within the TG and brains, thus apparently validating the trigeminal nerve route of viral transmission (Jeong et al., 2022). Based on these early reports, we conducted a pilot experiment investigating the olfactory nerve route of neuroinvasion to determine the regions of greatest viral tropism within the brains of hACE2 transgenic mice; it was during this pilot experiment that we incidentally found that the TG was intensely infected as well. In more recent studies, TG infection by SARS-CoV-2 has also been reported in deer mice (Fagre et al., 2021). However, these previous studies failed to thoroughly investigate the trigeminal nerve route of neuroinvasion by SARS-CoV-2 and illustrate the implications of this route of SARS-CoV-2 neuroinvasion on COVID-19 pathogenesis in great detail. Based on these early findings and our observations in the preliminary exploratory experiment, we present here our thorough and detailed findings confirming the trigeminal nerve route as an early and efficient route of SARS-CoV-2 neuroinvasion in hACE2 transgenic mice, thereby resulting in a highly neurovirulent and neurotropic viral infection that induces altered neural function without obvious neuroinflammation or cell death.

## Materials and methods

All procedures involving animals and infectious virus were performed in a biosafety level 3 (BSL-3) or animal biosafety level 3 (ABSL-3) facility at Galveston National Laboratory at the University of Texas Medical Branch (UTMB) at Galveston, Texas, an Association for Assessment and Accreditation of Laboratory Animal Care (AAALAC)-accredited (November 24, 2020) and Public Health Service-Office of Laboratory Animal Welfare (PHS-OLAW)-approved (February 26, 2021) high-containment National Laboratory. All animal procedures were carried out in accordance with animal protocols approved by an Institutional Animal Care and Use Committee (IACUC) at UTMB.

### Virus

The SARS-CoV-2 (strain US-WA-1/2020) used throughout this study was generously provided to us by Dr. Natalie Thornburg at the Centers for Disease Control (CDC), Atlanta, GA, through the World Reference Center for Emerging Viruses and Arboviruses (WRCEVA). SARS-CoV-2 were propagated in Vero-E6 cells maintained in Eagle’s Minimal Essential Medium (MEM) (Corning, 10-010-CV) supplemented with 2% fetal bovine serum (FBS), 2% L-Glutamine (GIBCO, 25030-164), and 1% Penicillin–Streptomycin (GIBCO, 15140-122); this media formulation has been designated “2-MEM.” The original stock of SARS-CoV-2 was cultured in 2-MEM and passaged two more times in Vero-E6 cells to generate the working viral stocks, which were stored at − 80°C. The working viral stocks used throughout this study were titrated at ~5 × 10^6^ TCID_50_/mL by a standard TCID_50_ assay in Vero-E6 cells.

### Mice

AC70 hACE2 transgenic mice were obtained from Taconic Biosciences, Inc. (Germantown, New York, United States). Initially generated in our laboratory in response to the 2003 SARS outbreak, AC70 hACE2 transgenic mice were previously extensively characterized in our laboratory for both SARS-CoV-1 and SARS-CoV-2 (Drelich et al., 2024; Tseng et al., 2007; Yoshikawa et al., 2009).

### Cells

Vero-E6 immortalized African green monkey kidney cells (CRL-1580, American Type Culture Collection) were grown in a media formulation designated “10-MEM,” a media formulation similar to 2-MEM but supplemented instead with 10% FBS.

### SARS-CoV-2 infection and necropsy

Isoflurane-anesthetized female AC70 hACE2 transgenic mice at 8–9 weeks old were challenged intranasally with 1 × 10^3^ TCID_50_ SARS-CoV-2 in 60 μL of 2-MEM; five control mice were mock-challenged with the same volume of phosphate-buffered saline. All mice were weighed daily to monitor disease progression. Additionally, illness severity in infected mice was scored independently by two investigators who used a standardized 1–4 grading system as follows: 1, healthy; 2, ruffled fur, lethargic; 3, ruffled fur, lethargic, hunched posture, orbital tightening, labored breathing/dyspnea, and/or more than 15% weight loss; 4, reluctance to move when stimulated or at least 20% weight loss. Each day after infection, five infected mice were sacrificed to obtain whole skulls for determining viral infectivity titers, staining for viral antigen by IHC as well as other antigens by two-color IF, profiling inflammatory responses, and histopathological analysis. The control mice were sacrificed on the first day post-mock-challenge (1 dpi) to harvest the same as above described. The whole skull samples were then split into left and right hemispheres, with the left hemispheres subsequently being further split into whole brain and trigeminal ganglion samples; half of each of whole brain and trigeminal ganglion samples were saved for viral yield determination while the other half of each tissue type samples would be homogenized in TRIzol (Invitrogen, Waltham, Massachusetts, United States) using TissueLyser-QIAGEN (Retsch, Haan, Germany). The clarified lysates of all homogenates were used for RNA extraction and RT-qPCR. The remaining right hemisphere of each whole skull sample was then fixed by immersion in 10%-buffered formalin for 72 h followed by transfer to 70% ethanol.

### Determination of tissue viral yields

Brain and trigeminal ganglion samples were homogenized in 2% FBS-PBS using TissueLyser-QIAGEN; homogenates were then clarified via low-speed centrifugation. Clarified lysates were subjected to viral titration (TCID_50_).

### End-point dilution median tissue culture infectious dose viral titration assay

The end-point dilution median tissue culture infectious dose (TCID_50_) viral titration assay was performed as previously described (Tseng et al., 2007; Yoshikawa et al., 2009; Yap et al., 2021). Briefly summarized, we carried out a 1:10 serial dilution from 10^−1^ to 10^−8^ from a starting dilution of 50 μL of viral samples into 450 μL of 2-MEM. Then, we aliquoted 100 μL of the dilution into a 96-well plate of confluent Vero E6 cells at four wells each dilution. All 96-well plates were incubated at 37°C at 5% CO_2_ for up to 3 days, after which the number of wells exhibiting cytopathic effect were counted for each dilution. Then, the number of viable virions were calculated and quantified based on the Reed and Muench method and expressed as TCID_50_/mL (Reed and Muench, 1938).

### RNA extraction and reverse transcription-quantitative polymerase chain reaction

Total RNA was isolated from the tissues of infected mice homogenized in TRIzol solution as indicated above using a chloroform extraction method according to manufacturer instructions. Contaminating genomic DNA was removed upon digestion with DNase I during the extraction procedure using a DNase I clean-up kit (Invitrogen, AM1907, Waltham, Massachusetts, United States). The resulting RNA samples were subjected to two-step RT-qPCR analysis to assess the expression of SARS-CoV-2 E gene as well as other genes, starting with reverse transcription into cDNA using the iScript Reverse Transcription kit (Bio-Rad, 1,708,841, Hercules, California, United States). The primers for all genes can be seen in Table 1. 18S rRNA was used as the endogenous control. 20 ng cDNA was amplified for each replicate, with each animal specimen being assayed in duplicate for each gene, using an iTaq Universal SYBR Green supermix reagent kit (BioRad, 1,725,124, Hercules, California, United States), in a CFX96 thermocycler (BioRad, Hercules, California, United States). The cycling parameters for PCR for 40 cycles were as follows: initial polymerase activation at 95°C for 30 s, denaturation at 95°C for 10 s, and annealing/extension with plate read at 60°C for 30 s. The relative fold gene expression for each sample was calculated based on the Livak delta–delta Ct method (Livak and Schmittgen, 2001).

**Table 1:** List of RT-qPCR primers used in this study.

Gene	Forward primer (5′-3′)	Reverse primer (5′-3′)
*18 s*	GGACCAGAGCGAAAGCATTTGCC	TCAATCTCGGGTGGCTGAACG
*Sars2e*	ACAGGTACGTTAATAGTTAATAGCGT	ATATTGCAGCAGTACGCACACA
*Ifnα*	GACCTTCCTCAGACTCATAACC	CATCCACCTTCTCCTGCG
*Ifnβ*	GCGGACTTCAAGATCCCTATG	ACAATAGTCTCATTCCACCCAG
*Ifnγ*	AAATCCTGCAGAGCCAGATTAT	GCTGTTGCTGAAGAAGGTAGTA
*Tnfα*	TTGTCTACTCCCAGGTTCTCT	GAGGTTGACTTTCTCCTGGTATG
*Il1β*	TGGACCTTCCAGGATGAGGACA	GTTCATCTCGGAGCCTGTAGTG
*Il6*	TACCACTTCACAAGTCGGAGGC	CTGCAAGTGCATCATCGTTGTTC
*Il10*	AGCCGGGAAGACAATAACTG	GGAGTCGGTTAGCAGTATGTTG
*Il4*	TTGAGAGAGATCATCGGCATTT	CTCACTCTCTGTGGTGTTCTTC
*Ip10*	ATCATCCCTGCGAGCCTATCCT	GACCTTTTTTGGCTAAACGCTTTC
*Mcp1*	GTCCCTGTCATGCTTCTGG	GCTCTCCAGCCTACTCATTG
*Mx1*	TGGACATTGCTACCACAGAGGC	TTGCCTTCAGCACCTCTGTCCA
*Rantes*	CCTGCTGCTTTGCCTACCTCTC	ACACACTTGGCGGTTCCTTCGA
*Drd1*	TCTGGTTTACCTGATCCCTCA	GCCTCCTCCCTCTTCAGGT
*Th*	GCCAAGGACAAGCTCAGGAA	CTCAGTGCTTGGGTCAGGGT
*Nefl*	GCGCCATGCAGGACACA	ACCTGGCCATCTCGCTCTT
*Eno2*	AGGTGGATCTCTATACTGCCAAA	GTCCCCATCCCTTAGTTCCAG
*Syn1a*	AGCTCAACAAATCCCAGTCTCT	CGGATGGTCTCAGCTTTCAC
*Snap25*	CAACTGGAACGCATTGAGGAA	GGCCACTACTCCATC CTGATTAT

### Histopathology and immunostaining

Formalin-fixed whole skull sections in 70% ethanol from the above-described necropsy were subsequently paraffin-embedded and then sectioned at 5 μm thickness along the sagittal plane. Histopathological evaluation was performed on deparaffinized sections stained by routine hematoxylin-and-eosin (H&E) staining. Testing for the SARS-CoV-2 S viral antigen was performed using a standard colorimetric indirect horseradish peroxidase (HRP) IHC protocol modified from a previously described protocol (Tseng et al., 2007; Yoshikawa et al., 2009) using a rabbit anti-SARS-CoV-2 S protein antibody (Abcam, ab272504, Cambridge, United Kingdom) at 1:5000 dilution (0.2 μg/mL). Heat-mediated antigen retrieval at pH 6 using citrate buffer was performed. Specifically, the primary antibody was detected using the ImmPRESS^®^ HRP Horse Anti-Rabbit IgG PLUS Polymer Kit (Vector Laboratories, MP-7801-15, Newark, California, United States) following manufacturer instructions. Counterstaining was achieved with Mayer’s hematoxylin (Sigma-Aldrich, MHS16-500 mL, St. Louis, Missouri, United States). For two-color IF staining, the above IHC protocol was modified such that the anti-SARS-CoV-2 S primary antibody was at 1:1000 dilution (1 μg/mL) in background-reducing antibody diluent (Dako, S302283-2, Santa Clara, California, United States). A second primary antibody to detect a different cell marker antigen was simultaneously used with the SARS-CoV-2 S primary antibody at the following dilutions: TUBB3/Tuj1 (GeneTex, GTX85469, Irvine, California, United States, 1:100), GFAP (GeneTex, GTX85454, Irvine, California, United States, 1:500), and ACE2 (R&D Systems, AF933, Minneapolis, MN, United States, 1:250); IBA1 (GeneTex, GTX637629, Irvine, California, United States, 1:100) was used with a different mouse SARS-CoV-2 S antibody (GeneTex, GTX632604, Irvine, California, United States, 1:1000). The primary antibodies were then visualized using secondary antibodies conjugated with the appropriate listed fluorophores: goat anti-rabbit IgG Alexa Fluor 568 (Invitrogen, A-11011, Waltham, Massachusetts, United States, 1:1000), goat anti-chicken IgY Alexa Fluor 488 (Invitrogen, A-11039, Waltham, Massachusetts, United States, 1:2000), and goat anti-mouse IgG Alexa Fluor 555 (Invitrogen, A-32727, Waltham, Massachusetts, United States, 1:2000).

### Graph creation and statistical analysis

Statistical analysis was performed, and graphs were created, in GraphPad Prism 10.2.3. Student’s *t*-test was used for all statistical analyses.

## Results

### Intranasal challenge with a lethal dose of SARS-CoV-2 caused a profound infection in the TG before spreading to the brain

**Figure 1:**
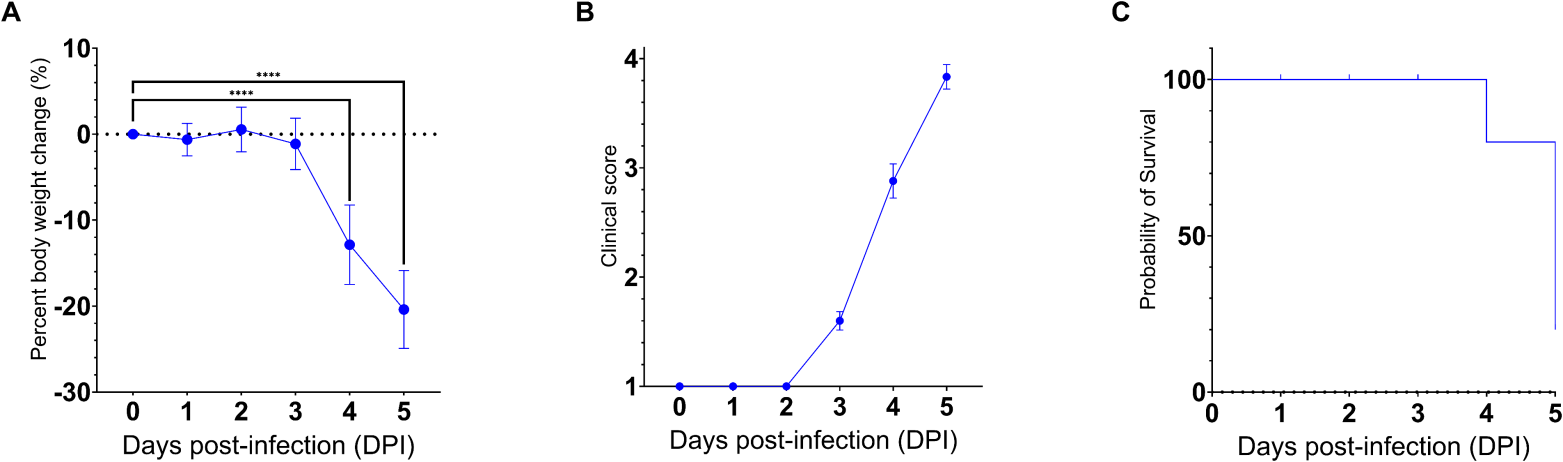
SARS-CoV-2 infection via the intranasal route results in a rapid development of clinical disease and mortality. 25 AC70 mice were intranasally inoculated with 1 × 10^3^ TCID_50_ of SARS-CoV-2 strain US-WA-1/2020 in 2% fetal bovine serum-supplemented cell media (2-MEM) and then five mice were sacrificed each day for 5 days post-infection. **(A)** Significant weight loss rapidly developed starting on 4 dpi, correlating with **(B)** the onset of severe clinical signs of disease. **(C)** The onset of clinical disease quickly advanced to near full mortality at 5 dpi Weight changes were expressed as the mean percent changes in infected animals relative to the initial weights at 0 dpi Error bars represent standard errors of the mean (SEM). ****p < 0.0001. These were the representative results of one out of two independent experiments.

To gain insights into the neuroinvasive potential of SARS-CoV-2, we intranasally challenged AC70 human ACE2 (hACE2) transgenic mice with 1 × 10^3^ TCID_50_ (approximately 333 LD_50_) of SARS-CoV-2 (US-WA-1/2020 strain) (Drelich et al., 2024; manuscript in press) and monitored them daily for morbidity (e.g., weight changes) and mortality. Starting on 4 days post-infection (dpi), the challenged mice began to exhibit significant weight loss as well as other signs of disease, before rapidly succumbing to infection with nearly 100% mortality by 5 dpi (Figures 1A–C). Then, we assessed the kinetics of viral spread within the brain and its nearby peripheral nervous structures, e.g., OE and TG, by using immunohistochemical (IHC) staining for SARS-CoV-2 Spike (S) protein.

**Figure 2:**
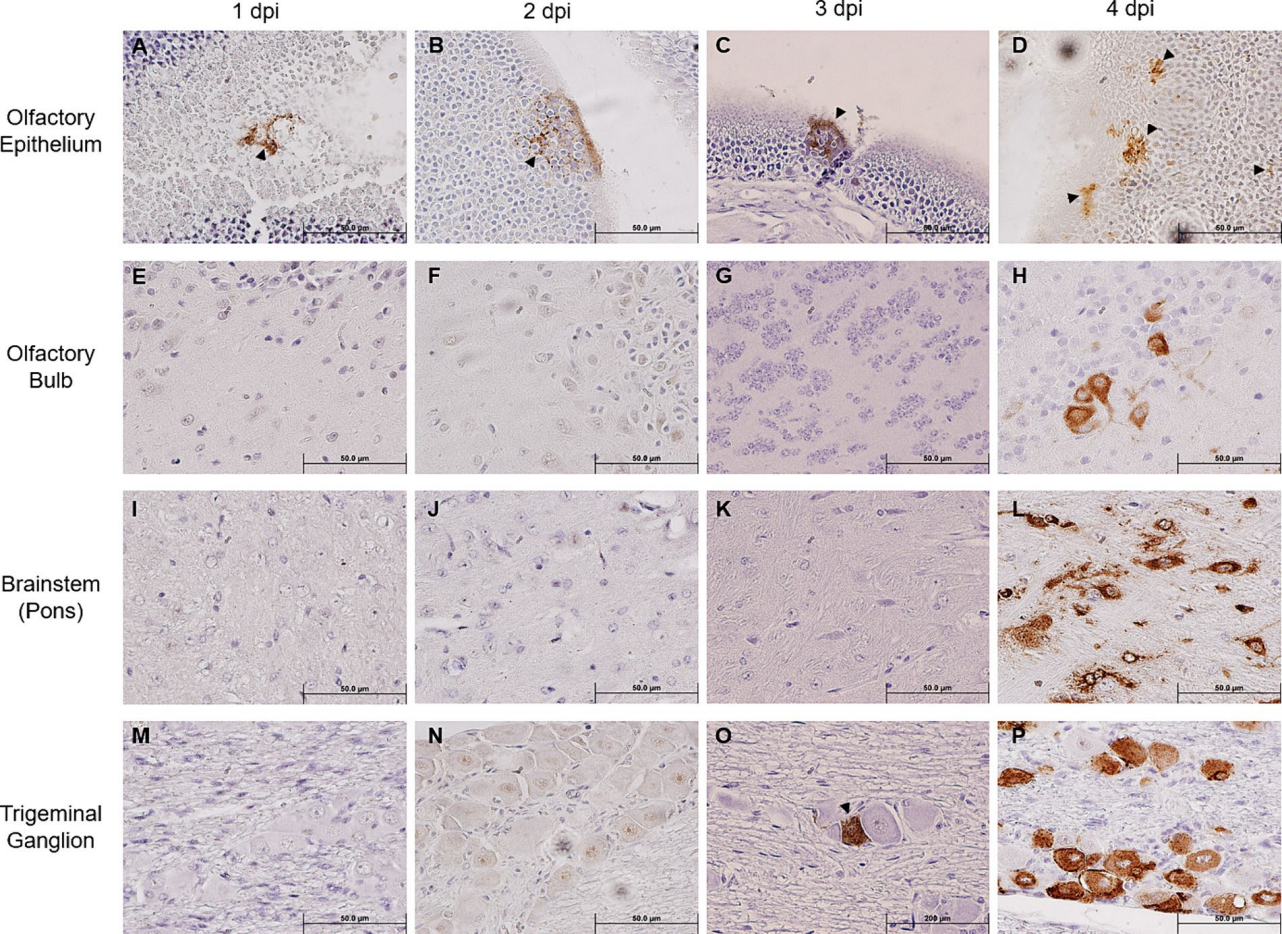
Immunohistochemical analysis of SARS-2 antigen in the brain, OE, and TG after infection via the intranasal route. Formalin-fixed, paraffin-embedded (FFPE) skull sagittal or coronal sections containing the brain, OE, and TG were analyzed via immunohistochemistry (IHC) for the expression of the SARS-2 spike (S) protein. SARS-2 S antigen (brown) can be detected only in the OE in one out of five mice (sole positive shown in **A**) starting from 1 dpi but can begin to be detected in the TG starting from 3 dpi; SARS-2 S could not be detected in all other regions of the brain from 1 dpi to 3 dpi, including the olfactory bulb. Black arrowheads indicate selected points of antigen detection. **(A–D)** Olfactory epithelium; **(E–H)** olfactory bulb; **(I–L)** brainstem (pons); **(M–P)** trigeminal ganglion. Magnification 40X. Blue nuclei indicate hematoxylin counter-staining.

We noted that SARS-CoV-2 S could only be sporadically detected within the OE as early as 1 dpi (one out of five mice), and progressively sustained thereafter through 4 dpi (Figures 2A–D), thereby confirming earlier reports that the OE serves as an early site of viral replication (Meinhardt et al., 2021; Zhang et al., 2020). Despite the incrementing viral infection of OE over time, we were unable to detect any SARS-CoV-2 S within the OB until 4 dpi (Figures 2E–H). Similarly, we could not detect any SARS-CoV-2 S staining within the pons either until 4 dpi (Figures 2I–L). An analysis revealed that at 4 dpi most challenged mice stained positively for SARS-CoV-2 S in the OB and the pons. Interestingly, we were able to unambiguously detect the expression of SARS-CoV-2 S in the TG starting at 3 dpi in a couple of infected mice, of which the staining intensity profoundly increased in all infected mice at 4 dpi (Figures 2M–P). Taken together, the finding of TG as a permissive site of SARS-CoV-2 infection suggests that the trigeminal nerve route could be another early and olfactory nerve-independent route of neuroinvasion by SARS-CoV-2 to enter the CNS/brain. Nevertheless, consistent with the onset of severe disease in infected mice, we observed at 4 dpi overwhelming viral infection in all major anatomic regions of the brain, including the proposed initial ports of entry, i.e., OB and pons of the olfactory nerve and trigeminal nerve neuroinvasive routes, respectively (Figures 2H,L), as well as regions distal to the initial sites of entry, such as the frontal cerebral cortex (prefrontal, somatomotor, somatosensory, etc.), basal ganglia (caudate putamen and striatum), thalamus, hypothalamus, hippocampal formation, cerebellum, and brainstem (mesencephalon and medulla) (Supplementary Figure S2). At 5 dpi, we observed the viral antigen staining continue to spread throughout almost all regions of the brain (Supplementary Figure S3).

### Kinetics of SARS-CoV-2 viral infection in brain and TG

**Figure 3:**
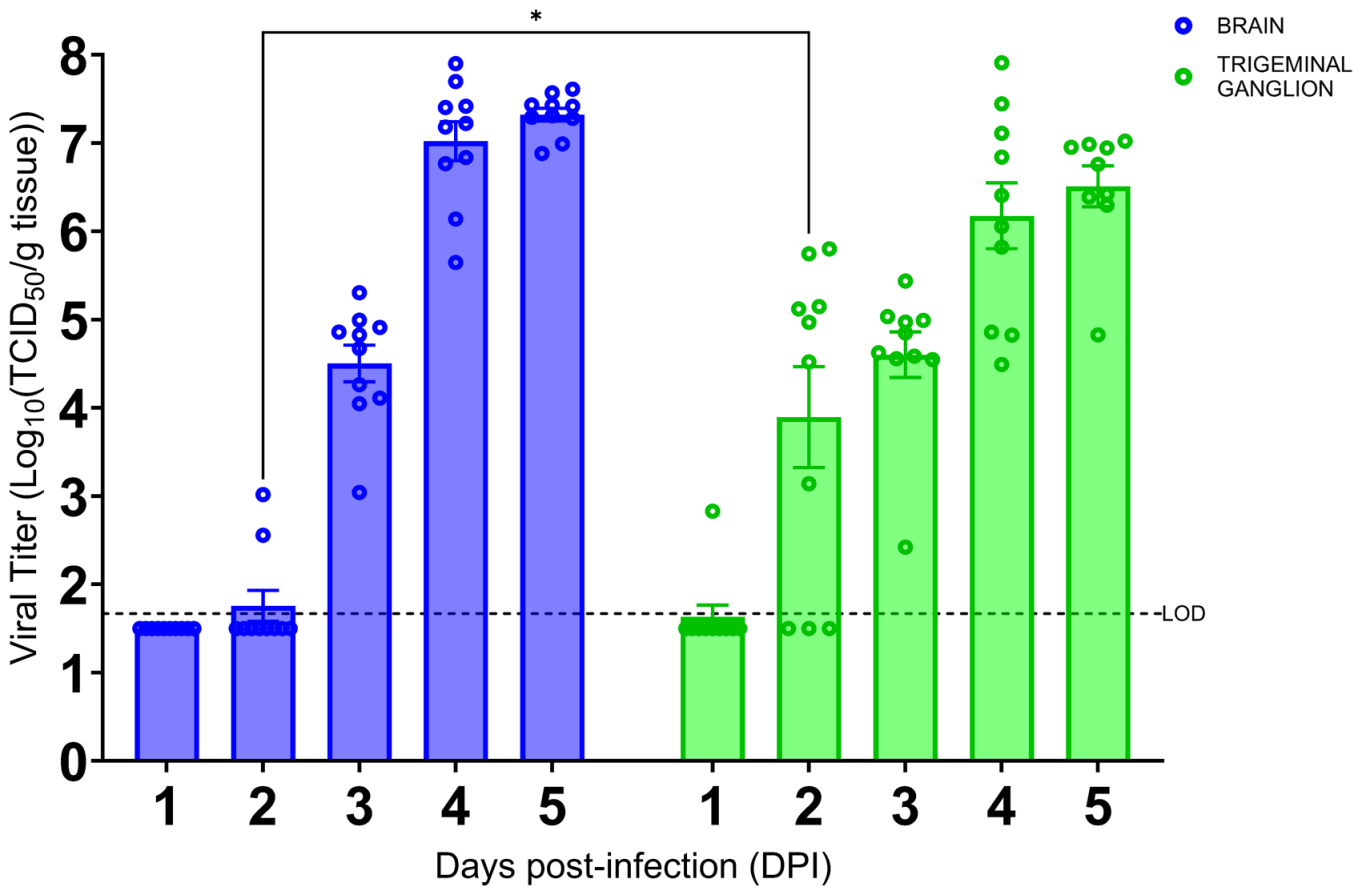
SARS-CoV-2 replication kinetics in the trigeminal ganglion and the brain. The titers of infectious virus in brain and TG were calculated and expressed as log_10_ TCID_50_ virus per gram of tissue and were plotted as the mean of two different cohorts (*n* = 10 animals per timepoint). Virus titers in the brain (blue) and TG (green) were assessed using a standard Vero-E6 cell-based TCID_50_ assay. * *p* < 0.05, by Student’s *t*-test, comparing brain and TG. Error bars represent standard errors of the mean (SEM). These were the combined data of two different independent experiments.

As we have shown the TG is an early site of SARS-CoV-2 infection, we investigated the kinetics of viral infection within the brain and TG. As shown in Figure 3, we found that infectious virus could be recovered from the brain at 3 dpi with a titer of approximately 4.5 log TCID_50_/g, followed by a sharp increase to ~7 log and ~ 7.5 log TCID_50_/g at 4 and 5 dpi, respectively. While we could not detect any signs of viral infection by IHC staining within the TG until 3 dpi (Figure 2O), infectious virus was recovered at 2 dpi (~4 log TCID_50_/g). The titers of infectious virus in the TG increased thereafter to 6.5 log TCID_50_/g 5 dpi. Specifically, while we were only able to isolate a low titer of live virus from the TG of 1/10 challenged mice at 1 dpi, we saw an increase in viral detection reaching 7/10 and 10/10 mice at 2 and 3 dpi and thereafter. Therefore, viral replication occurs 1 day earlier in the TG than in the brain.

### Neurons are the primary brain cells supporting productive SARS-CoV-2 infection

**Figure 4:**
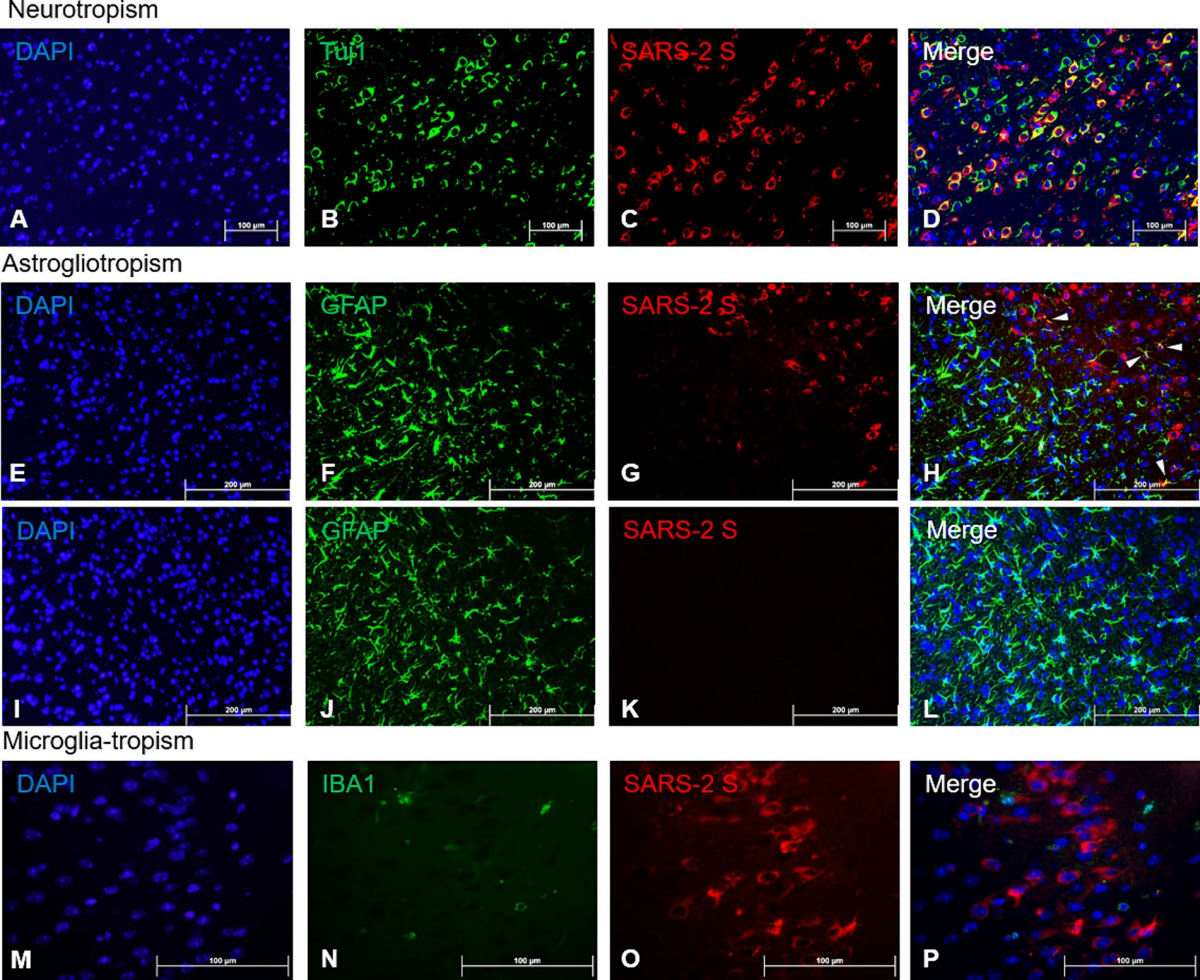
Viral tropism analysis via immunofluorescence of SARS-CoV-2 antigen in the brain. FFPE serial sections of the brain harvested at 4 dpi were analyzed via dual-labeling immunofluorescence (IF) for the expression of the SARS-2 spike (S) protein (red) and different cell identity markers (green). **(A–D)** Neurons (Tuj1^+^, frontal cortex); **(E–L)** Astrocytes (GFAP^+^, frontal cortex), white arrowheads indicate selected points of colocalization, **(E–H)** SARS-CoV-2-infected mice, **(I–L)** mock-infected mice; **(M–P)** Microglia (IBA1^+^, frontal cortex). Magnifications: **(A–L)**, 10X; **(M–P)**, 40X. DAPI counterstaining (blue).

The profound SARS-CoV-2 infection within the brain of infected mice prompted us to investigate the identity of permissive brain cells by using two-color immunofluorescent (IF) staining on 4 dpi sections by simultaneously targeting specific cell markers and viral antigens. Encouraged by the data shown in Figure 2 and Supplementary Figure S3 that a vast majority of cells positively stained for the S protein by IHC morphologically resembled neuronal cells, we repeated the IHC staining for the expression of beta tubulin III (TUBB3, also known as Tuj1), a marker of neuronal cells, and SARS-CoV-2 S protein. We found that most cells that were stained positively for the S protein co-expressed Tuj1 in the cytosols of the main bodies of the neurons, indicating neurons are likely the prime brain cells permissive to SARS-CoV-2 infection (Figures 4A–D). To further verify that neurons were indeed the preferred brain cells targeted by SARS-CoV-2, we use the same IHC staining technique for detecting the expressions of glial fibrillary acidic protein (GFAP) and ionized calcium-binding adaptor molecule 1 (IBA1), markers for astrocytes and microglia, respectively, along with SARS-CoV-2 S protein. While there were a few cells co-labelled with GFAP and SARS-CoV-2 S (Figure 4H, arrowheads), the majority of GFAP^+^ astroglia were not permissive to SARS-CoV-2 infection (Figures 4E–H). Moreover, we did not observe any signs of proliferative response (astrogliosis) and activation of astroglia (Figure 4F), based on the absence of detectable extension and thickening of cellular processes (Pekny et al., 2014; Pekny and Pekna, 2014; Garman, 2011), when compared to mock-infected animals (Figure 4J). In contrast to SARS-CoV-2-permissive neurons and, astroglia, to a much lesser extent, we were unable to reveal any cells dually labelled with IBA1, the marker of microglial cells, and SARS-CoV-2 S protein (Figures 4M–P), indicating that microglial cells likely are not permissive to infection by SARS-CoV-2. However, as we could only detect very few cells that were IBA1^+^, we could not make any conclusions on microglial activation based on proliferation and retraction of processes (Figure 4N).

**Figure 5:**
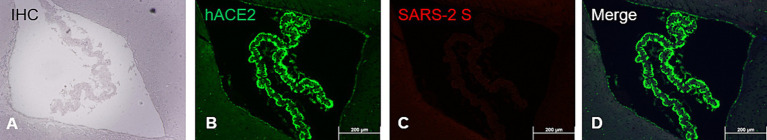
ACE2 co-expression with SARS-CoV-2 S antigen at the choroid plexus. FFPE brain section showing specifically the choroid plexus analyzed via immunostaining (IHC and dual-labeling IF) for hACE2 and SARS-2 S. **(A)** SARS-2 S IHC (brown); **(B)** hACE2 IF (ACE2^+^, green); **(C)** SARS-2 S IF (SARS-2 S^+^, red); **(D)** Merge of hACE2 and SARS-2 IF (ACE2^+^ and SARS-2 S^+^). Magnification 10X. IHC hematoxylin counterstaining (blue).

While human (h) ACE2 transgene is known to constitutively express in tissues/organs of AC70 transgenic mice (Tseng et al., 2007; Yoshikawa et al., 2009), to what extent this human hACE2 expression conferred the susceptibility of brain cells to SARS-CoV-2 infection has not been fully investigated. To study this, paraffin-embedded brain sections of infected AC70 mice were subjected to the standard IHC staining for hACE2 and SARS-CoV-2 S protein, as described above. As shown in Figures 5A–D, we found that in the choroid plexus (ChP), hACE2 expression alone cannot act as the determinant for permissiveness to SARS-CoV-2 infection; the ChP, which is composed of endothelial and glial ependymal cells, was shown to intensely express hACE2 (Figure 5B), which is consistent with earlier reports (Chen et al., 2021), and yet, cells within this region apparently were not stained positively with SARS-CoV-2 S protein (Figures 5C,D). Furthermore, we found that the expression of hACE2 in the TG was comparable to that in the rest of the brain, suggesting that the early detection of viral infection within the TG was not dependent on the relative expression levels of hACE2 (Supplementary Figure S4). Whether the lack of infection is caused by a lack of additional host factors, such as TMPRSS2, or that the choroid plexus might be physically anatomically secluded from the virus as a result of its unique anatomic location remain currently unknown and warrant additional investigation.

### Host responses to SARS-CoV-2 infection within the brains of AC70 transgenic mice

**Figure 6:**
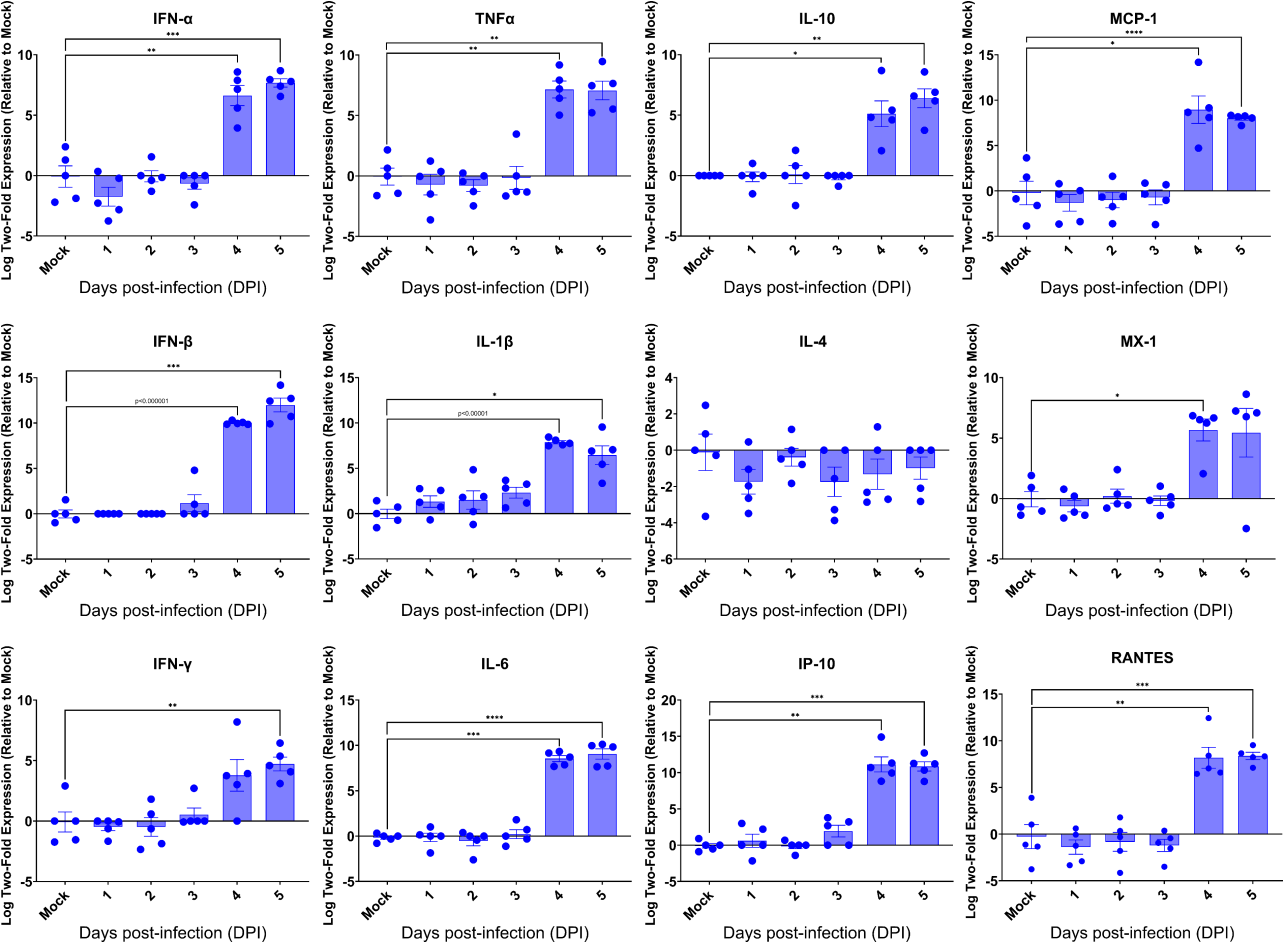
Kinetics of the cytokine responses in the brains of SARS-2-infected AC70 mice. Total RNA extracted from the brains of AC70 mice sacrificed daily after SARS-2 infection were used to measure the expression of various cytokines and chemokines by RT-qPCR. Each individual brain sample was assayed in duplicate. Results are shown as the mean for five animals at each time point. Error bars represent SEM. * *p* < 0.05, ** *p* < 0.01, *** *p* < 0.001, **** *p* < 0.0001, *p* < 0.00001 where indicated (Student’s *t*-test, compared to mock-infected mice).

Having revealed the profound viral infection throughout major anatomical regions of the brain, we investigated how host would respond in the brain upon lethal challenge with SARS-CoV-2. We initially profiled the inflammatory responses by RT-qPCR, followed by examining the brain sections for the histopathology. We found that among 13 inflammatory mediators measured, 11 mediators were significantly induced in the brains at 4 dpi (Figure 6), the time when significantly elevated viral titers were recovered, as shown in Figure 3. We also found that 10 out of 11 virally induced soluble mediators were proinflammatory, including IFN-I (*α*/*β*), IFN-II (*γ*), TNF-α, IL-1β, IL-6, IP-10, MCP-1, MX-1, and RANTES, whereas the transcriptional levels of IL-4 and IL-10, markers of Th2 and anti-inflammatory or inflammatory regulator, respectively, were either slightly downregulated (IL-4) or significantly upregulated (IL-10). Despite the significant expression of inflammatory mediators within the brain, we did not identify any infiltrates of mononuclear cells within H&E-stained sections of the brains even at 4 dpi (Supplementary Figure S1). Because SARS-CoV-2 infection exhibits cytopathic effects resulting in the deaths of permissive host cells (Ogando et al., 2020), we examined the brain sections for any histopathological signs of cell death. As shown in Supplementary Figure S1, we were unable to detect any signs of apoptotic cell death as apoptotic cell deaths were the most common type of cell deaths associated with neurovirulent viral infections. We used the standard TUNEL assay kit (Abcam, Cambridge, United Kingdom) to detect apoptotic cells within the brains harvested at 5 dpi. Among a total of five brains examined, only one exhibited a few apoptotic cells while the other four were negative for TUNEL assays (data not shown). Together, these results suggested that infected brain cells, especially neurons as indicated by the IF dual-labeling results (Figures 4A–D), but not inflammatory infiltrates, are the likely sources of the inflammatory mediators detected in the brain. Additionally, the lack of inflammatory infiltrates and convincing cell death within the brain emphasize the neuronopathy, but not encephalitis, is the likely cause of death of acutely infected AC70 transgenic mice.

### Neuroinvasive SARS-CoV-2 infection dysregulated the expression of key genes regulating neurological functions

**Figure 7:**
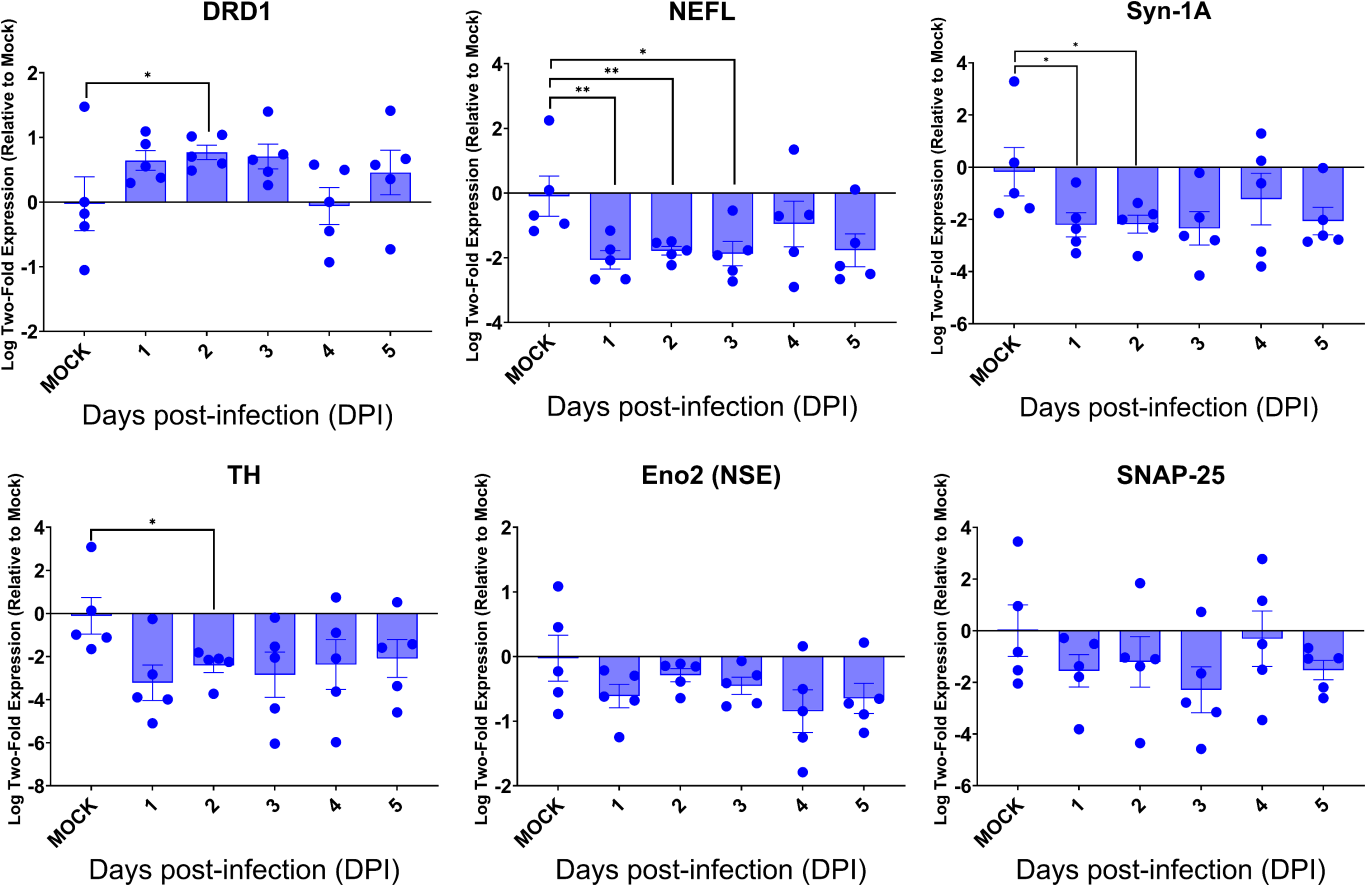
SARS-CoV-2 brain infection alters gene expression of neuronal function and neural damage markers. Total RNA extracted from the brains of infected AC70 mice daily after SARS-2 infection were used to measure the gene expression levels of selected neural biomarkers of damage (*Nefl* and *Eno2*) and neuronal function (*DRD1*, *TH*, *Syn-1A*, and *SNAP-25*). Each individual brain sample was assayed in duplicate. Results are shown as means (±SEM) of five animals at each time point. * *p* < 0.05, ** *p* < 0.01 (Student’s *t*-test, compared to mock-infected mice).

Despite the intense SARS-CoV-2 infection within the brain, preferentially targeting neurons, especially neurons, of acutely infected AC70 transgenic mice that succumbed to infection within days, we did not reveal histopathological evidence of neuroinflammatory response (encephalitis) with noticeable cell death. We examined whether this seemingly nonlytic, but extensive neuronal infection of SARS-CoV-2 might still alter neurological functions. Thus, we explored the expression of six key genes, i.e., *DRD1*, *TH*, *NEFL*, *Eno2* (*NSE*), *Syn-1A*, *SNAP-25*, that serve as the regulators and markers for neuronal function and damage, respectively, in the brains of infected AC70 transgenic mice over time, compared to uninfected brains. We found that, except for *DRD1*, the transcriptional expressions of all other five genes evaluated were downregulated, with statistical significance only at a few timepoints for *TH*, *NEFL*, and *Syn-1A* (Figure 7). The up-and down-regulated expressions of the dopamine receptor D1 (*DRD1*) and tyrosine hydroxylase (*TH*), respectively, a functional pair of molecules governing a unique neuronal function, is of interest; at 2 dpi, the significant upregulation of DRD1 was mirrored by the significant downregulation of TH (Figure 7). Intriguingly, the kinetics of gene expression do not reflect the kinetics of viral replication. While the exact nature of the lack of any direct correlation between viral infection and dysregulated gene expression remains unknown, our results might suggest that the impact on neuron transmission/gene expression may be initiated before viral infection could be readily detected. Regardless, our findings are novel in specifically connecting SARS-CoV-2 infection to an immediate impact on neurological functions. Nevertheless, these results show that neuroinvasive SARS-CoV-2 infection could indeed alter functional gene expression without causing neuroinflammation or obvious cell death.

## Discussion

In the study of coronavirus pathogenesis and particularly that of SARS-CoV-2, the trigeminal nerve is a possible route into the CNS that is often overlooked in favor of the olfactory nerve. Widely reported and generally accepted as the main route of entry into the CNS, the olfactory nerve route alone does not explain how SARS-CoV-2 infection could penetrate the main bulk of the brain at its rear (Bulfamante et al., 2021; Bulfamante et al., 2020; Emmi et al., 2023). To this end, we mapped and characterized the trigeminal nerve route of neuroinvasion by SARS-CoV-2 in the AC70 hACE2 transgenic mouse model and found that there are many potential consequences. Specifically, we demonstrated that the trigeminal nerve may be an early and highly efficient site of SARS-CoV-2 viral replication, on par with that of the olfactory nerve, and that SARS-CoV-2 viral infection primarily targets neurons, leading to changes in neural function with minimal tissue pathology.

Early in the course of viral infection, SARS-CoV-2 can be readily detected in the TG, even before the onset of weight loss and disease signs in our mouse model. IHC staining showing the early SARS-CoV-2 S staining in the TG (Figures 2O,P), supported by the high viral titer of the TG at an earlier timepoint (Figure 3), implied that viral infection of the trigeminal nerve occurred nearly simultaneously with the OE and olfactory nerve, which would have occurred immediately after intranasal challenge. Additionally, the SARS-CoV-2 S staining pattern changing from undetectable to observable in all major anatomic regions of the brain within the span of 1 day suggested that the spread of viral infection occurred extremely rapidly. Since on 4 dpi the peripheral regions of the brain closest to both the OE (e.g., OB and frontal cortex) and the TG (e.g., hypothalamus, midbrain, and pons) (Supplementary Figure S3A) stained more intensely with SARS-CoV-2 S than the central internal regions, we speculated that SARS-CoV-2 neuroinvasion proceeded via retrograde axonal transmission. Alternatively, the more extensive viral antigen staining of the peripheral anatomic regions of the brain than the central regions could indicate the virus was transported in the cerebrospinal fluid (CSF), as has been previously suggested (Viszlayová et al., 2021); not only is the TG itself situated anatomically in the CSF-rich Meckel’s cavity, but terminals of all three branches of the trigeminal nerve penetrate through the cribriform plate to innervate skull bone marrow niches and ultimately the dura mater of the meninges, which are awash in CSF (Kamel and Toland, 2001; Kemp et al., 2012; Pulous et al., 2022). Although the TG and trigeminal nerve were previously reported to have been infected early on during the heights of the COVID-19 pandemic (Meinhardt et al., 2021), this study greatly extends the findings from the previous study by investigating the dynamics of SARS-CoV-2 neuroinvasion along the trigeminal nerve on a time course basis.

Our findings indicate that the primary target of SARS-CoV-2 viral tropism in the brain is neurons, but not astrocytes, microglia, or even endothelial cells. Initially, based on the expression levels of ACE2, in descending order, the main targets of SARS-CoV-2 tropism in the brain were thought to be endothelial cells, and then ependymal glial cells, astrocytes, and microglia, but not neurons (Brann et al., 2020; Hamming et al., 2004). Yet, our IF staining showing the amount of SARS-CoV-2 S expression in different cell types to be highest in neurons (Figures 4A–D) suggests otherwise in our model. We identified an anatomic structure of the brain, the ChP, as not only the structure of the brain with one of the highest expression levels of ACE2 as previously reported (Chen et al., 2021; Pellegrini et al., 2020), but one that was not observably infected by SARS-CoV-2 at all. We were interested in the permissiveness of the ChP to SARS-CoV-2 because the ChP has been suggested as an alternative portal of viral entry into the brain via the hematogenous routes due to its composition of primarily endothelial and ependymal glial cells (Song et al., 2021; Chen et al., 2021; Pellegrini et al., 2020).

Despite the profound viral infection throughout the brain, the brain was notably devoid of the typical correlates of tissue pathology associated with neuro-dysfunction. Although the pro-inflammatory cytokines were generally significantly induced corresponding to the magnitude and kinetics of viral replication, we were initially very surprised to observe an overall lack of any histopathological signs of neuroinflammation; inflammatory infiltrates and the associated vascular cuffing were rarely, if at all, observed, while our IF staining revealed a lack of astrocytic and microglial activation, which is atypical for viral neuroinvasion. However, we rationalized this by considering a sufficiently highly virulent and rapid viral infection has the ability to induce an immunosuppressive state that the neuroimmune system does not have the time to mount an inflammatory response before the organism succumbs (Borrow et al., 1995; Libbey and Fujinami, 2002; McChesney et al., 1989). Additionally, we noticed that the occurrences of apoptosis were not consistent with the vast extent of viral infection; for example, in two anatomic regions of the brain with heavy viral infection, the frontal cortex and medulla oblongata, there were only a few cells actively undergoing apoptosis as revealed by TUNEL assay, while the hypothalamus, one of the most heavily infected region of the brain, had no cells detected undergoing apoptosis. However, we again surmised that owing to their postmitotic nature and limited numbers, mature neurons are remarkably resistant to apoptosis and programmed cell death, even after viral infections (Hollville et al., 2019; Yakovlev and Faden, 2004; Kole et al., 2013). All the histopathological results combined prompted us to investigate the expression levels of neural function markers, namely those for dopamine neurotransmitter processing and synaptic function, to gain any insights into the mechanisms of neurodysfunction. The pair of DRD1 and TH is relevant because this gene couple is directly involved in neurotransmitter dopamine processing; TH is the rate-limiting enzyme that catalyzes the production of the dopamine precursor L-DOPA (Daubner et al., 2011), while DRD1 is the most abundant dopamine receptor within the CNS (Zhuang et al., 2021). Furthermore, the downregulation of the neural damage markers neurofilament light chain (*NEFL*) and neuron-specific enolase (*NSE*) (Figure 7) ran counter to our expectations; in cases of acute brain injury, such as traumatic brain injuries (TBI) or ischemic events, NEFL and NSE have been reported to be elevated as markers of neuronal injury (Kim et al., 2018; Graham et al., 2021; Shahim et al., 2016; Lee et al., 2021; Rech et al., 2006). These results indicated to us that there were indeed alterations to neural function because of viral infection, whether due to inflammation or viral infection. Our results suggest that there are alternative mechanisms of neurological disorder that extend beyond neuroinflammation and cell death.

The neuroinvasive potential of SARS-CoV-2 has been a controversial topic, with some reports suggesting the non-permissiveness of neurons to SARS-CoV-2 and limited invasion of the CNS (Butowt et al., 2021; Thakur et al., 2021; Pedrosa et al., 2021; Jagst et al., 2024), but more recent studies have reported contradictory findings. SARS-CoV-2 has been reported to infect neurons and has in fact been found in the trigeminal ganglia of human patients (Song et al., 2021; Meinhardt et al., 2021; Emmi et al., 2023; Stein et al., 2022). Although brain tissue damage caused by SARS-CoV-2 infection has been reported, other studies have reported the lack of direct viral infection-induced tissue damage in line with our data (Matschke et al., 2020; Seehusen et al., 2022; Stein et al., 2023). *In vivo* studies using the K18-hACE2 mouse model of SARS-CoV-2 neuroinvasion are in line with our findings such as viral tropism for neurons as well as minimal histopathological changes in the infected brains (Seehusen et al., 2022; Oladunni et al., 2020). Moreover, despite the fact that hACE2 transgenic mouse lines, such as AC70 and K18-hACE2, and other established mouse and other animal models of SARS-CoV-2 infection, may not fully recapitulate the pathogenesis of COVID-19 and pattern of ACE2 expression in human patients, the findings of this study still bring insights into the neuropathogenic mechanisms of SARS-CoV-2 infection.

**Figure 8:**
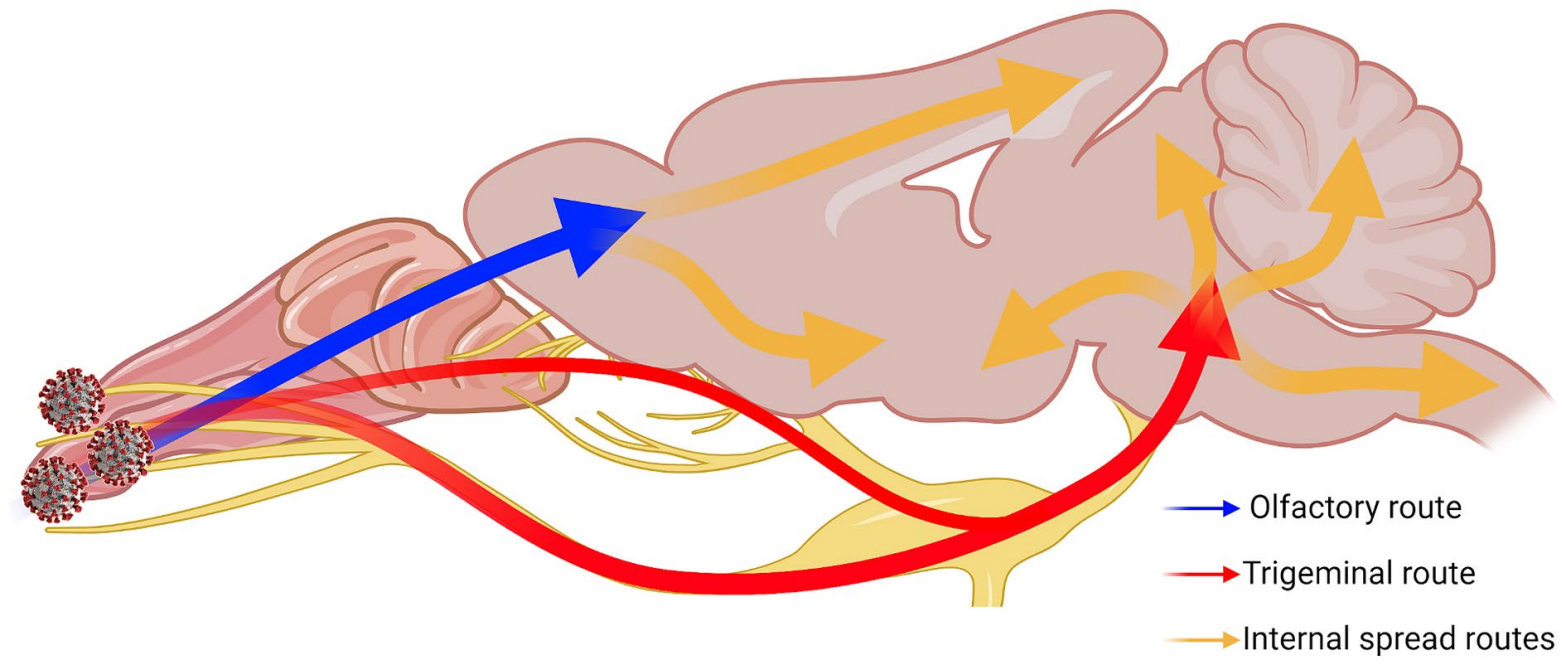
Two-pronged neuroinvasion route of SARS-CoV-2. Theorized two-pronged neuroinvasion route taken by SARS-CoV-2 starting from the nasal cavity during respiratory infection based on the observed viral antigen staining pattern. Red and blue arrows indicate the two main forks invading into the brain [e.g., OE → OB (blue); nasal/olfactory epithelium → TG → pons (red)]. Orange arrows indicate speculated routes of further viral spread inside the brain proper once brain has been penetrated. Created with Biorender.com.

Overall, our results show that the trigeminal nerve is an early and efficient site of SARS-CoV-2 infection in our model, suggesting that it may be an efficient entry route to the brain/CNS. Based on all our results together, we speculated neuroinvasive SARS-CoV-2 uses a two-pronged route from the OB and the pons towards the center of the brain (Figure 8). We also characterized alterations in neuronal function that were observed despite a general lack of typical histopathological findings of neuroinflammation. It is clear from these findings that additional studies are warranted for the further characterization of COVID-19 neuropathogenesis.

## Data Availability

The raw data supporting the conclusions of this article will be made available by the authors, without undue reservation.

## Supplementary material

SUPPLEMENTARY FIGURE S1 Histopathological analysis of SARS-CoV-2-infected mice brains. Micrograph of whole skull section (right hemisphere) of infected AC70 mouse at 5 dpi show a lack of abnormalities or differences between the mock-infected sections **(A–D)** and sections from the SARS-2-infected brains **(E–H)**. Magnification 10X.

SUPPLEMENTARY FIGURE S2 Immunohistochemical analysis of SARS-CoV-2 antigen in the brain at 4 dpi. Images of FFPE skull sections of infected AC70 mice at 4 dpi. **(A)** Whole left hemisphere, 10X; **(B)** pre-frontal cortex, 40X; **(C)** striatum, 40X; **(D)** thalamus, 40X; **(E)** hypothalamus, 40X; **(F)** hippocampus, 40X; **(G)** cerebellum, 40X; **(H)** medulla oblongata, 40X. Insets’ origins marked by lower-case letters in **(A)**. Blue nuclei indicate hematoxylin counter-staining.

SUPPLEMENTARY FIGURE S3 Immunohistochemical analysis of SARS-CoV-2 antigen in the brain at 5 dpi. Micrograph of whole skull section (right hemisphere) of infected AC70 mouse at 5 dpi.

SUPPLEMENTARY FIGURE S4 Protein expression via immunofluorescence of ACE2 in the brain and trigeminal ganglion. Infected AC70 mice at 5 dpi.

## References

[ref1] Al-Aly Z. Xie Y. Bowe B. (2021). High-dimensional characterization of post-acute sequelae of COVID-19. Nature 594, 259–264. doi: 10.1038/s41586-021-03553-9, PMID: 33887749

[ref2] Arbour N. Day R. Newcombe J. Talbot P. J. (2000). Neuroinvasion by human respiratory coronaviruses. J. Virol. 74, 8913–8921. doi: 10.1128/JVI.74.19.8913-8921.2000, PMID: 10982334 PMC102086

[ref3] Baig A. M. Sanders E. C. (2020). Potential neuroinvasive pathways of SARS-CoV-2: deciphering the spectrum of neurological deficit seen in coronavirus disease-2019 (COVID-19). J. Med. Virol. 92, 1845–1857. doi: 10.1002/jmv.26105, PMID: 32492193 PMC7300748

[ref4] Bauer L. Laksono B. M. de Vrij F. M. S. Kushner S. A. Harschnitz O. van Riel D. (2022). The neuroinvasiveness, neurotropism, and neurovirulence of SARS-CoV-2. Trends Neurosci. 45, 358–368. doi: 10.1016/j.tins.2022.02.006, PMID: 35279295 PMC8890977

[ref5] Beier K. T. (2021). The serendipity of viral trans-neuronal specificity: more than meets the eye. Front. Cell. Neurosci. 15:720807. doi: 10.3389/fncel.2021.720807 34671244 PMC8521040

[ref6] Borrow P. Evans C. F. Oldstone M. B. (1995). Virus-induced immunosuppression: immune system-mediated destruction of virus-infected dendritic cells results in generalized immune suppression. J. Virol. 69, 1059–1070. doi: 10.1128/jvi.69.2.1059-1070.1995, PMID: 7815484 PMC188677

[ref7] Brann D. H. Tsukahara T. Weinreb C. Lipovsek M. Van den Berge K. Gong B.. (2020). Non-neuronal expression of SARS-CoV-2 entry genes in the olfactory system suggests mechanisms underlying COVID-19-associated anosmia. Sci. Adv. 6:eabc5801. doi: 10.1126/sciadv.abc5801 32937591 PMC10715684

[ref8] Bulfamante G. Bocci T. Falleni M. Campiglio L. Coppola S. Tosi D.. (2021). Brainstem neuropathology in two cases of COVID-19: SARS-CoV-2 trafficking between brain and lung. J. Neurol. 268, 4486–4491. doi: 10.1007/s00415-021-10604-8, PMID: 34003372 PMC8129960

[ref9] Bulfamante G. Chiumello D. Canevini M. P. Priori A. Mazzanti M. Centanni S.. (2020). First ultrastructural autoptic findings of SARS-Cov-2 in olfactory pathways and brainstem. Minerva Anestesiol. 86, 678–679. doi: 10.23736/S0375-9393.20.14772-2, PMID: 32401000

[ref10] Butowt R. Meunier N. Bryche B. von Bartheld C. S. (2021). The olfactory nerve is not a likely route to brain infection in COVID-19: a critical review of data from humans and animal models. Acta Neuropathol. 141, 809–822. doi: 10.1007/s00401-021-02314-2, PMID: 33903954 PMC8075028

[ref11] Chen R. Wang K. Yu J. Howard D. French L. Chen Z.. (2021). The spatial and cell-type distribution of SARS-CoV-2 receptor ACE2 in the human and mouse brains. Front. Neurol. 11:573095. doi: 10.3389/fneur.2020.573095, PMID: 33551947 PMC7855591

[ref12] Coronaviridae Study Group of the International Committee on Taxonomy of Viruses (2020). The species severe acute respiratory syndrome-related coronavirus: classifying 2019-nCoV and naming it SARS-CoV-2. Nat. Microbiol. 5, 536–544. doi: 10.1038/s41564-020-0695-z, PMID: 32123347 PMC7095448

[ref13] Daubner S. C. Le T. Wang S. (2011). Tyrosine hydroxylase and regulation of dopamine synthesis. Arch. Biochem. Biophys. 508, 1–12. doi: 10.1016/j.abb.2010.12.017, PMID: 21176768 PMC3065393

[ref14] Desforges M. Coupanec A. Le Brison É. Meessen-Pinard M. Talbot P. J., editors. Neuroinvasive and neurotropic human respiratory coronaviruses: potential Neurovirulent agents in humans (2014); New Delhi: Springer India. 10.1007/978-81-322-1777-0_6 PMC7121612 24619619

[ref15] Dong E. Du H. Gardner L. (2020). An interactive web-based dashboard to track COVID-19 in real time. Lancet Infect. Dis. 20, 533–534. doi: 10.1016/S1473-3099(20)30120-1, PMID: 32087114 PMC7159018

[ref16] DosSantos M. F. Devalle S. Aran V. Capra D. Roque N. R. Coelho-Aguiar J. M.. (2020). Neuromechanisms of SARS-CoV-2: a review. Front. Neuroanat. 14:37. doi: 10.3389/fnana.2020.00037 32612515 PMC7308495

[ref17] Drelich A. K. Rayavara K. Hsu J. Saenkham-Huntsinger P. Judy B. M. Tat V.. (2024). Characterization of unique pathological features of COVID-associated coagulopathy: studies with AC70 hACE2 transgenic mice highly permissive to SARS-CoV-2 infection. PLoS Pathog. 20:e1011777. doi: 10.1371/journal.ppat.1011777, PMID: 38913740 PMC11226087

[ref18] Emmi A. Rizzo S. Barzon L. Sandre M. Carturan E. Sinigaglia A.. (2023). Detection of SARS-CoV-2 viral proteins and genomic sequences in human brainstem nuclei. NPJ Parkinsons Dis. 9:25. doi: 10.1038/s41531-023-00467-3 36781876 PMC9924897

[ref19] Fagre A. Lewis J. Eckley M. Zhan S. Rocha S. M. Sexton N. R.. (2021). SARS-CoV-2 infection, neuropathogenesis and transmission among deer mice: implications for spillback to New World rodents. PLoS Pathog. 17:e1009585. doi: 10.1371/journal.ppat.1009585, PMID: 34010360 PMC8168874

[ref20] Garman R. H. (2011). Histology of the central nervous system. Toxicol. Pathol. 39, 22–35. doi: 10.1177/0192623310389621 21119051

[ref21] Graham N. S. N. Zimmerman K. A. Moro F. Heslegrave A. Maillard S. A. Bernini A.. (2021). Axonal marker neurofilament light predicts long-term outcomes and progressive neurodegeneration after traumatic brain injury. Sci. Transl. Med. 13:eabg9922. doi: 10.1126/scitranslmed.abg9922 34586833

[ref22] Hamming I. Timens W. Bulthuis M. L. Lely A. T. Navis G. van Goor H. (2004). Tissue distribution of ACE2 protein, the functional receptor for SARS coronavirus. A first step in understanding SARS pathogenesis. J. Pathol. 203, 631–637. doi: 10.1002/path.1570 15141377 PMC7167720

[ref23] Hoffmann M. Kleine-Weber H. Pöhlmann S. (2020). A multibasic cleavage site in the spike protein of SARS-CoV-2 is essential for infection of human lung cells. Mol. Cell 78, 779–84.e5. doi: 10.1016/j.molcel.2020.04.022, PMID: 32362314 PMC7194065

[ref24] Hoffmann M. Kleine-Weber H. Schroeder S. Kruger N. Herrler T. Erichsen S.. (2020). SARS-CoV-2 cell entry depends on ACE2 and TMPRSS2 and is blocked by a clinically proven protease inhibitor. Cell 181, 271–280.e8. doi: 10.1016/j.cell.2020.02.052, PMID: 32142651 PMC7102627

[ref25] Hollville E. Romero S. E. Deshmukh M. (2019). Apoptotic cell death regulation in neurons. FEBS J. 286, 3276–3298. doi: 10.1111/febs.14970, PMID: 31230407 PMC6718311

[ref26] Huang Y. H. Jiang D. Huang J. T. (2020). SARS-CoV-2 detected in cerebrospinal fluid by PCR in a case of COVID-19 encephalitis. Brain Behav. Immun. 87:149. doi: 10.1016/j.bbi.2020.05.012, PMID: 32387508 PMC7202824

[ref27] Jagst M. Pottkämper L. Gömer A. Pitarokoili K. Steinmann E. (2024). Neuroinvasion and neurotropism of severe acute respiratory syndrome coronavirus 2 infection. Curr. Opin. Microbiol. 79:102474. doi: 10.1016/j.mib.2024.102474, PMID: 38615394

[ref28] Jeong G. U. Kwon H.-J. Ng W. H. Liu X. Moon H. W. Yoon G. Y.. (2022). Ocular tropism of SARS-CoV-2 in animal models with retinal inflammation via neuronal invasion following intranasal inoculation. Nat. Commun. 13:7675. doi: 10.1038/s41467-022-35225-1, PMID: 36509737 PMC9743116

[ref29] Jeong G. U. Lyu J. Kim K.-D. Chung Y. C. Yoon G. Y. Lee S.. (2022). SARS-CoV-2 infection of microglia elicits Proinflammatory activation and apoptotic cell death. Microbiol. Spectr. 10, e01091–e01022. doi: 10.1128/spectrum.01091-22 35510852 PMC9241873

[ref30] Kamel H. A. M. Toland J. (2001). Trigeminal nerve anatomy. Am. J. Roentgenol. 176, 247–251. doi: 10.2214/ajr.176.1.1760247 11133576

[ref31] Kemp W. J. Tubbs R. S. Cohen-Gadol A. A. (2012). The innervation of the cranial dura mater: neurosurgical case correlates and a review of the literature. World Neurosurg. 78, 505–510. doi: 10.1016/j.wneu.2011.10.045, PMID: 22120554

[ref32] Kim H. J. Tsao J. W. Stanfill A. G. (2018). The current state of biomarkers of mild traumatic brain injury. JCI Insight 3:e97105. doi: 10.1172/jci.insight.97105 29321373 PMC5821170

[ref33] Kole A. J. Annis R. P. Deshmukh M. (2013). Mature neurons: equipped for survival. Cell Death Dis. 4:e689. doi: 10.1038/cddis.2013.220 23807218 PMC3702294

[ref34] Lee D. Cho Y. Ko Y. Heo N. H. Kang H. G. Han S. (2021). Neuron-specific enolase level as a predictor of neurological outcome in near-hanging patients: a retrospective multicenter study. PLoS One 16:e0246898. doi: 10.1371/journal.pone.0246898, PMID: 33566872 PMC7875384

[ref35] Libbey J. E. Fujinami R. S. (2002). “Virus-induced immunosuppression” in Polymicrobial diseases. ed. Brogden K. A. G. J. (Washington (DC): ASM Press). 21735561

[ref36] Livak K. J. Schmittgen T. D. (2001). Analysis of relative gene expression data using real-time quantitative PCR and the 2−ΔΔCT method. Methods 25, 402–408. doi: 10.1006/meth.2001.1262 11846609

[ref37] Matschke J. Lütgehetmann M. Hagel C. Sperhake J. P. Schröder A. S. Edler C.. (2020). Neuropathology of patients with COVID-19 in Germany: a post-mortem case series. Lancet Neurol. 19, 919–929. doi: 10.1016/S1474-4422(20)30308-2, PMID: 33031735 PMC7535629

[ref38] McChesney M. B. Fujinami R. S. Lerche N. W. Marx P. A. Oldstone M. B. A. (1989). Virus-induced immunosuppression: infection of peripheral blood mononuclear cells and suppression of immunoglobulin synthesis during natural measles virus infection of Rhesus monkeys. J. Infect. Dis. 159, 757–760. doi: 10.1093/infdis/159.4.757, PMID: 2784472

[ref39] Meinhardt J. Radke J. Dittmayer C. Franz J. Thomas C. Mothes R.. (2021). Olfactory transmucosal SARS-CoV-2 invasion as a port of central nervous system entry in individuals with COVID-19. Nat. Neurosci. 24, 168–175. doi: 10.1038/s41593-020-00758-5, PMID: 33257876

[ref40] Morris M. Zohrabian V. M. (2020). Neuroradiologists, be mindful of the Neuroinvasive potential of COVID-19. Am. J. Neuroradiol. 41, E37–E39. doi: 10.3174/ajnr.A6551 32354715 PMC7342739

[ref41] Natoli S. Oliveira V. Calabresi P. Maia L. F. Pisani A. (2020). Does SARS-Cov-2 invade the brain? Translational lessons from animal models. Eur. J. Neurol. 27, 1764–1773. doi: 10.1111/ene.14277, PMID: 32333487 PMC7267377

[ref42] Ogando N. S. Dalebout T. J. Zevenhoven-Dobbe J. C. Limpens R. W. A. L. van der Meer Y. Caly L.. (2020). SARS-coronavirus-2 replication in Vero E6 cells: replication kinetics, rapid adaptation and cytopathology. J. Gen. Virol. 101, 925–940. doi: 10.1099/jgv.0.001453, PMID: 32568027 PMC7654748

[ref43] Oladunni F. S. Park J.-G. Pino P. A. Gonzalez O. Akhter A. Allué-Guardia A.. (2020). Lethality of SARS-CoV-2 infection in K18 human angiotensin-converting enzyme 2 transgenic mice. Nat. Commun. 11:6122. doi: 10.1038/s41467-020-19891-7, PMID: 33257679 PMC7705712

[ref44] Paniz-Mondolfi A. Bryce C. Grimes Z. Gordon R. E. Reidy J. Lednicky J.. (2020). Central nervous system involvement by severe acute respiratory syndrome coronavirus-2 (SARS-CoV-2). J. Med. Virol. 92, 699–702. doi: 10.1002/jmv.25915, PMID: 32314810 PMC7264598

[ref45] Pedrosa C. S. G. Goto-Silva L. Temerozo J. R. Souza L. R. Q. Vitória G. Ornelas I. M.. (2021). Non-permissive SARS-CoV-2 infection in human neurospheres. Stem Cell Res. 54:102436. doi: 10.1016/j.scr.2021.102436, PMID: 34186311 PMC8236004

[ref46] Pekny M. Pekna M. (2014). Astrocyte reactivity and reactive Astrogliosis: costs and benefits. Physiol. Rev. 94, 1077–1098. doi: 10.1152/physrev.00041.2013 25287860

[ref47] Pekny M. Wilhelmsson U. Pekna M. (2014). The dual role of astrocyte activation and reactive gliosis. Neurosci. Lett. 565, 30–38. doi: 10.1016/j.neulet.2013.12.071, PMID: 24406153

[ref48] Pellegrini L. Albecka A. Mallery D. L. Kellner M. J. Paul D. Carter A. P.. (2020). SARS-CoV-2 infects the brain choroid plexus and disrupts the blood-CSF barrier in human brain organoids. Cell Stem Cell 27, 951–61.e5. doi: 10.1016/j.stem.2020.10.001, PMID: 33113348 PMC7553118

[ref49] Proal A. D. VanElzakker M. B. (2021). Long COVID or post-acute sequelae of COVID-19 (PASC): an overview of biological factors that may contribute to persistent symptoms. Front. Microbiol. 12:698169. doi: 10.3389/fmicb.2021.698169, PMID: 34248921 PMC8260991

[ref50] Ptaszyńska-Sarosiek I. Dunaj J. Zajkowska A. Niemcunowicz-Janica A. Król M. Pancewicz S.. (2019). Post-mortem detection of six human herpesviruses (HSV-1, HSV-2, VZV, EBV, CMV, HHV-6) in trigeminal and facial nerve ganglia by PCR. PeerJ 6:e6095. doi: 10.7717/peerj.6095, PMID: 30643675 PMC6330031

[ref51] Pulous F. E. Cruz-Hernández J. C. Yang C. Kaya Ζ. Paccalet A. Wojtkiewicz G.. (2022). Cerebrospinal fluid can exit into the skull bone marrow and instruct cranial hematopoiesis in mice with bacterial meningitis. Nat. Neurosci. 25, 567–576. doi: 10.1038/s41593-022-01060-2, PMID: 35501382 PMC9081225

[ref52] Rech T. H. Vieira S. R. Nagel F. Brauner J. S. Scalco R. (2006). Serum neuron-specific enolase as early predictor of outcome after in-hospital cardiac arrest: a cohort study. Crit. Care 10:R133. doi: 10.1186/cc5046, PMID: 16978415 PMC1751053

[ref53] Reed L. J. Muench H. (1938). A SIMPLE METHOD OF ESTIMATING FIFTY PER CENT ENDPOINTS12. Am. J. Epidemiol. 27, 493–497. doi: 10.1093/oxfordjournals.aje.a118408

[ref54] Romano N. Federici M. Castaldi A. (2019). Imaging of cranial nerves: a pictorial overview. Insights Imaging 10:33. doi: 10.1186/s13244-019-0719-5, PMID: 30877408 PMC6420596

[ref55] Schaefer M. L. Böttger B. Silver W. L. Finger T. E. (2002). Trigeminal collaterals in the nasal epithelium and olfactory bulb: a potential route for direct modulation of olfactory information by trigeminal stimuli. J. Comp. Neurol. 444, 221–226. doi: 10.1002/cne.10143, PMID: 11840476

[ref56] Seehusen F. Clark J. J. Sharma P. Bentley E. G. Kirby A. Subramaniam K.. (2022). Neuroinvasion and Neurotropism by SARS-CoV-2 variants in the K18-hACE2 mouse. Viruses 14:1020. doi: 10.3390/v14051020, PMID: 35632761 PMC9146514

[ref57] Shahim P. Gren M. Liman V. Andreasson U. Norgren N. Tegner Y.. (2016). Serum neurofilament light protein predicts clinical outcome in traumatic brain injury. Sci. Rep. 6:36791. doi: 10.1038/srep36791, PMID: 27819296 PMC5098187

[ref58] Shimeld C. Efstathiou S. Hill T. (2001). Tracking the spread of a lacZ-tagged herpes simplex virus type 1 between the eye and the nervous system of the mouse: comparison of primary and recurrent infection. J. Virol. 75, 5252–5262. doi: 10.1128/JVI.75.11.5252-5262.2001 11333907 PMC114931

[ref59] Solomon T. (2021). Neurological infection with SARS-CoV-2 — the story so far. Nat. Rev. Neurol. 17, 65–66. doi: 10.1038/s41582-020-00453-w, PMID: 33414554 PMC7789883

[ref60] Solomon I. H. Normandin E. Bhattacharyya S. Mukerji S. S. Keller K. Ali A. S.. (2020). Neuropathological features of Covid-19. N. Engl. J. Med. 383, 989–992. doi: 10.1056/NEJMc2019373, PMID: 32530583 PMC7304421

[ref61] Song E. Zhang C. Israelow B. Lu-Culligan A. Prado A. V. Skriabine S.. (2021). Neuroinvasion of SARS-CoV-2 in human and mouse brain. J. Exp. Med. 218:e20202135. doi: 10.1084/jem.20202135, PMID: 33433624 PMC7808299

[ref62] Stein J. A. Kaes M. Smola S. Schulz-Schaeffer W. J. (2023). Neuropathology in COVID-19 autopsies is defined by microglial activation and lesions of the white matter with emphasis in cerebellar and brain stem areas. Front. Neurol. 14:1229641. doi: 10.3389/fneur.2023.1229641, PMID: 37521293 PMC10374362

[ref63] Stein S. R. Ramelli S. C. Grazioli A. Chung J.-Y. Singh M. Yinda C. K.. (2022). SARS-CoV-2 infection and persistence in the human body and brain at autopsy. Nature 612, 758–763. doi: 10.1038/s41586-022-05542-y, PMID: 36517603 PMC9749650

[ref64] Thakur K. T. Miller E. H. Glendinning M. D. Al-Dalahmah O. Banu M. A. Boehme A. K.. (2021). COVID-19 neuropathology at Columbia University Irving medical center/New York Presbyterian hospital. Brain 144, 2696–2708. doi: 10.1093/brain/awab148, PMID: 33856027 PMC8083258

[ref65] Theil D. Derfuss T. Paripovic I. Herberger S. Meinl E. Schueler O.. (2003). Latent herpesvirus infection in human trigeminal ganglia causes chronic immune response. Am. J. Pathol. 163, 2179–2184. doi: 10.1016/S0002-9440(10)63575-4, PMID: 14633592 PMC1892378

[ref66] Tremblay C. Frasnelli J. (2018). Olfactory and trigeminal systems interact in the periphery. Chem. Senses 43, 611–616. doi: 10.1093/chemse/bjy049, PMID: 30052799

[ref67] Tseng C.-T. K. Huang C. Newman P. Wang N. Narayanan K. Watts D. M.. (2007). Severe acute respiratory syndrome coronavirus infection of mice transgenic for the human angiotensin-converting enzyme 2 virus receptor. J. Virol. 81, 1162–1173. doi: 10.1128/JVI.01702-06, PMID: 17108019 PMC1797529

[ref68] Viszlayová D. Sojka M. Dobrodenková S. Szabó S. Bilec O. Turzová M.. (2021). SARS-CoV-2 RNA in the cerebrospinal fluid of a patient with long COVID. Ther. Adv. Infect. Dis. 8:204993612110485. doi: 10.1177/20499361211048572 PMC8511908 34659752

[ref69] World Health Organization (2020). Pneumonia of unknown cause – China. Geneva, Switzerland: World Health Organization-Disease Outbreak News-Item.

[ref70] Yakovlev A. G. Faden A. I. (2004). Mechanisms of neural cell death: implications for development of neuroprotective treatment strategies. NeuroRx 1, 5–16. doi: 10.1602/neurorx.1.1.5, PMID: 15717003 PMC534908

[ref71] Yap T. F. Hsu J. C. Liu Z. Rayavara K. Tat V. Chien-Te K. T.. (2021). Efficacy and self-similarity of SARS-CoV-2 thermal decontamination. J. Hazard. Mater. 429:127709. doi: 10.1016/j.jhazmat.2021.127709 35086724 PMC8572375

[ref72] Yoshikawa N. Yoshikawa T. Hill T. Huang C. Watts D. M. Makino S.. (2009). Differential Virological and immunological outcome of severe acute respiratory syndrome coronavirus infection in susceptible and resistant transgenic mice expressing human angiotensin-converting enzyme 2. J. Virol. 83, 5451–5465. doi: 10.1128/JVI.02272-08, PMID: 19297479 PMC2681954

[ref73] Zhang A. J. Lee A. C. Chu H. Chan J. F. Fan Z. Li C.. (2020). SARS-CoV-2 infects and damages the mature and immature olfactory sensory neurons of hamsters. Clin. Infect. Dis. 73, e503–e512. doi: 10.1093/cid/ciaa995 PMC7454453 32667973

[ref74] Zhuang Y. Krumm B. Zhang H. Zhou X. E. Wang Y. Huang X.-P.. (2021). Mechanism of dopamine binding and allosteric modulation of the human D1 dopamine receptor. Cell Res. 31, 593–596. doi: 10.1038/s41422-021-00482-0, PMID: 33750903 PMC8089099

