# Supplementary figures and images for: Characterizing neuroinvasion and neuropathology of SARS-CoV-2 by using AC70 human ACE2 transgenic mice
Source: Front Microbiol. 2024 Sep 24;15:1455462. doi: 10.3389/fmicb.2024.1455462 (PMC11458418; doi:10.3389/fmicb.2024.1455462)

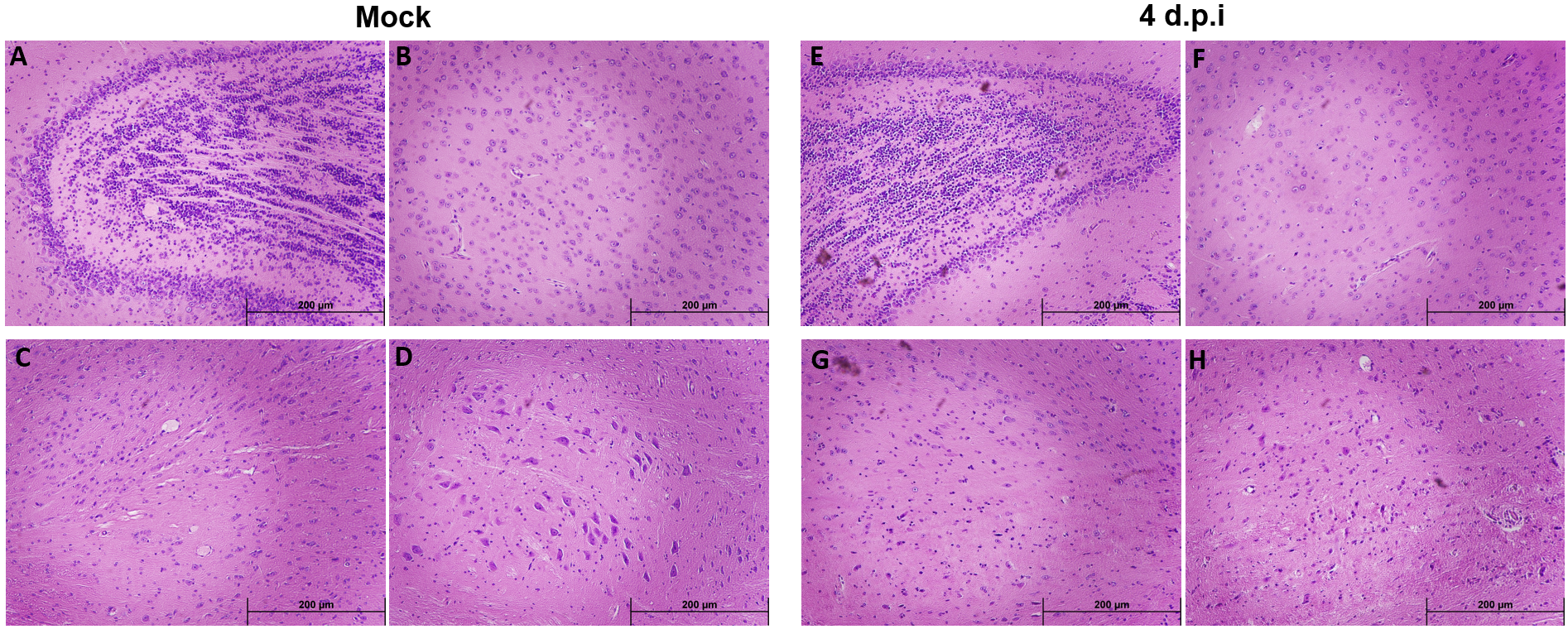

Supplement: SUPPLEMENTARY FIGURE S1 — Histopathological analysis of SARS-CoV-2-infected mice brains. Micrograph of whole skull section (right hemisphere) of infected AC70 mouse at 5 dpi show a lack of abnormalities or differences between the mock-infected sections (A–D) and sections from the SARS-2-infected brains (E–H). Magnification 10X. [file Image_1.TIF]

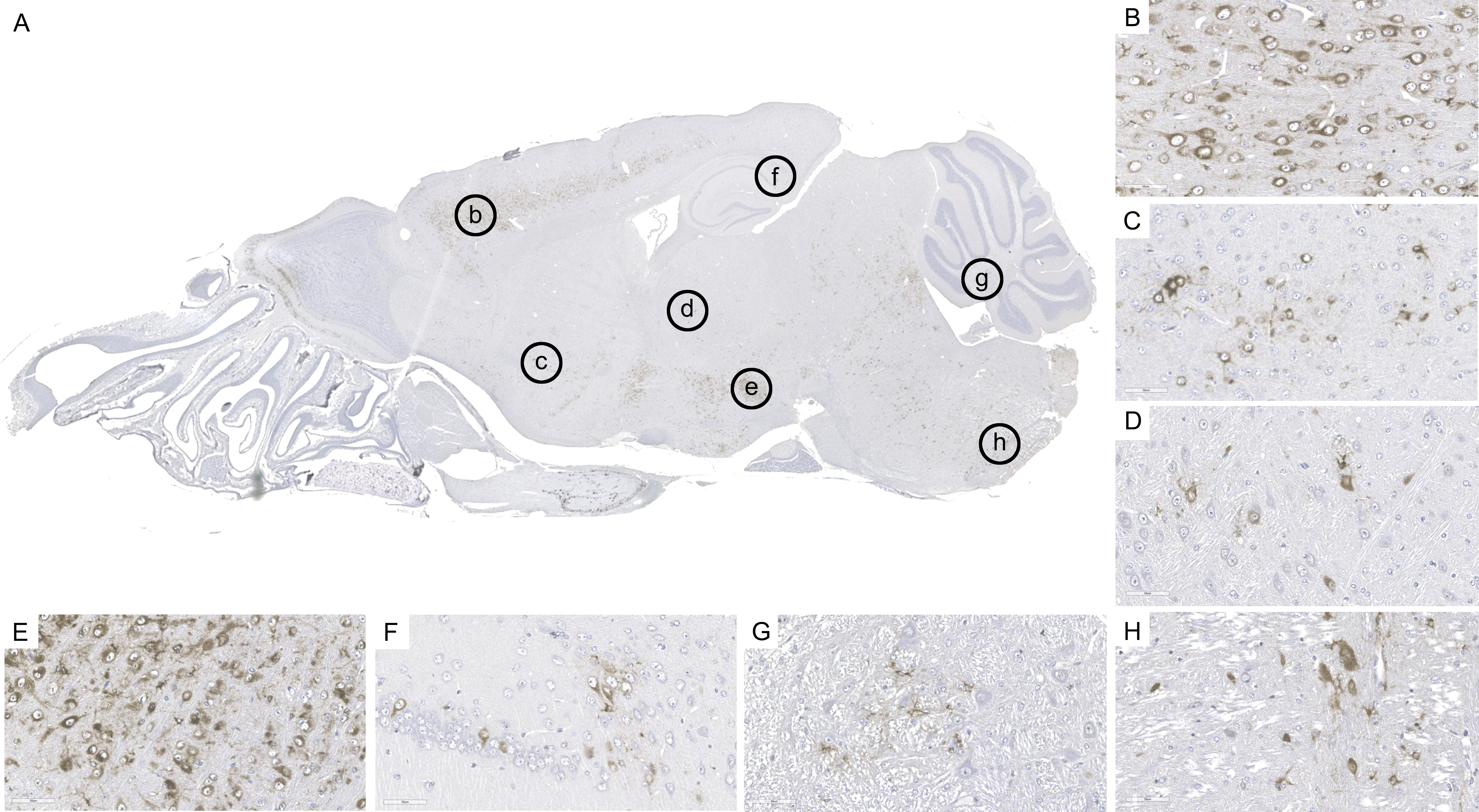

Supplement: SUPPLEMENTARY FIGURE S2 — Immunohistochemical analysis of SARS-CoV-2 antigen in the brain at 4 dpi. Images of FFPE skull sections of infected AC70 mice at 4 dpi. (A) Whole left hemisphere, 10X; (B) pre-frontal cortex, 40X; (C) striatum, 40X; (D) thalamus, 40X; (E) hypothalamus, 40X; (F) hippocampus, 40X; (G) cerebellum, 40X; (H) medulla oblongata, 40X. Insets’ origins marked by lower-case letters in (A). Blue nuclei indicate hematoxylin counter-staining. [file Image_2.TIF]

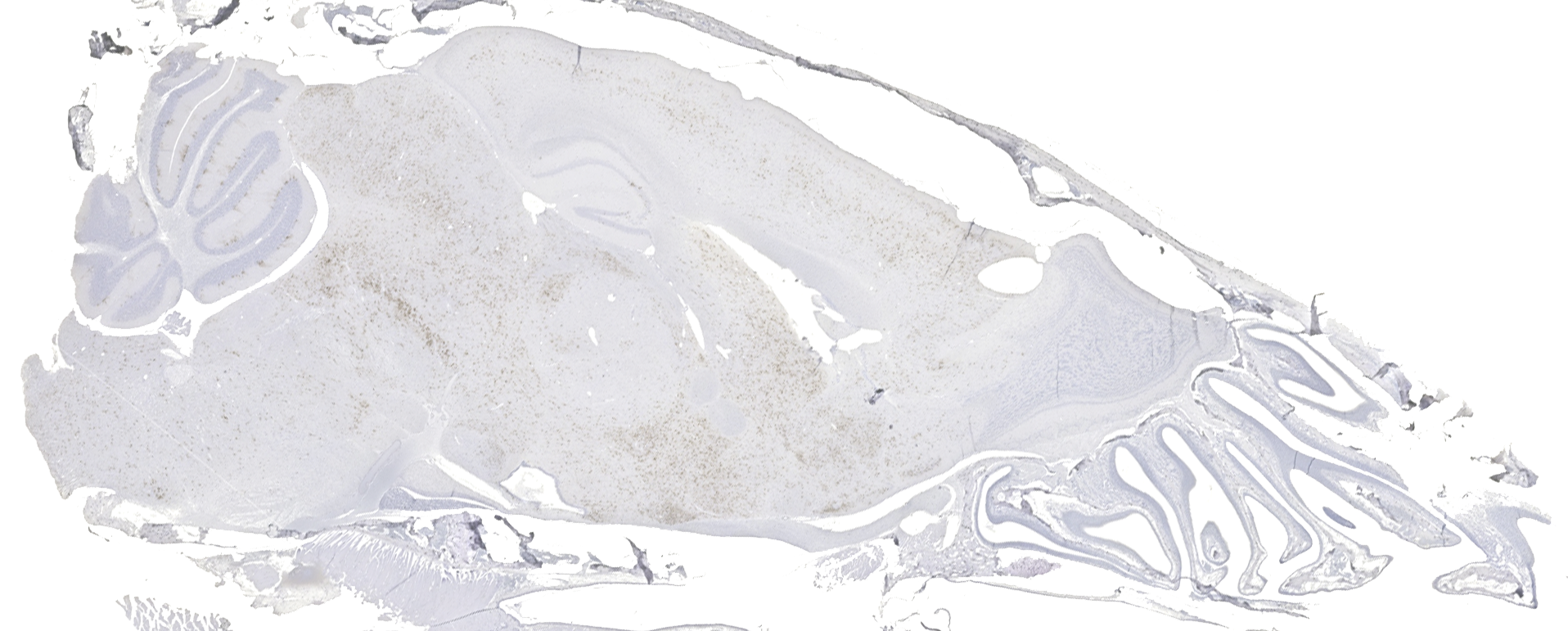

Supplement: SUPPLEMENTARY FIGURE S3 — Immunohistochemical analysis of SARS-CoV-2 antigen in the brain at 5 dpi. Micrograph of whole skull section (right hemisphere) of infected AC70 mouse at 5 dpi. [file Image_3.TIF]

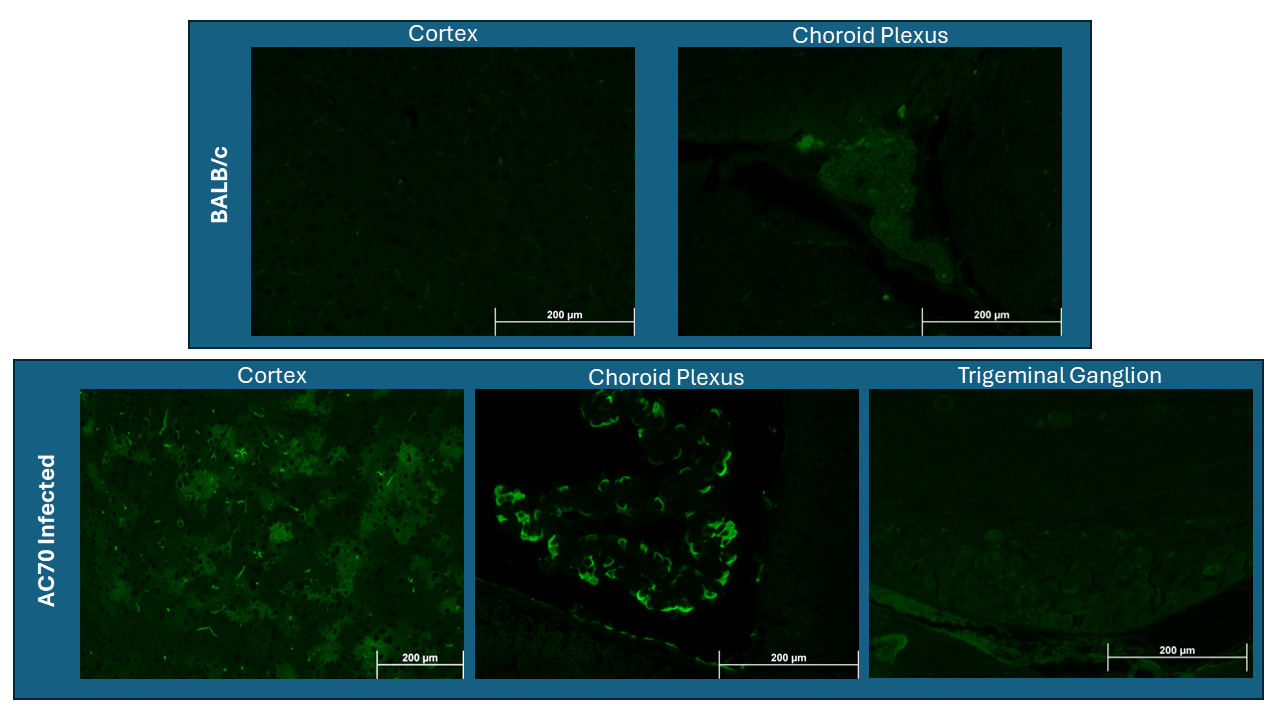

Supplement: SUPPLEMENTARY FIGURE S4 — Protein expression via immunofluorescence of ACE2 in the brain and trigeminal ganglion. Infected AC70 mice at 5 dpi. [file Image_4.TIF]
